# Supplementary material for: Information differences across spatial resolutions and scales for disease surveillance and analysis: The case of Visceral Leishmaniasis in Brazil
Source: PLoS One. 2020 Jul 17;15(7):e0235920. doi: 10.1371/journal.pone.0235920 (PMC7367469; doi:10.1371/journal.pone.0235920)
Supplement: Attachment — Submitted filename: Servadio_ResponseToReviewers.pdf [file pone.0235920.s004.pdf]

UNIVERSITY OF MINNESOTA

Twin Cities Campus

Division of Environmental Health Sciences
School of Public Health

Suite 1260 Mayo
420 Delaware street SE
Minneapolis, MN 55455

16 April 2020

Dear Dr. Carvalho and reviewers,

Thank you for taking the time to review our manuscript and for giving us the opportunity to submit a revised version. We thank the reviewers for the helpful feedback on our study. Our manuscript has been modified and improved based on the comments. Several points were clarified in the manuscript based on the reviewers' comments, including some methodological decisions and clarifications as well as some important topics for the discussion section.

The reviewer comments are seen italicized, and responses to specific comments are found directly below relevant comments, indented.

Reviewer #1*: This is a very relevant study that addresses the effect of spatial scales on visceral leishmaniasis surveillance data from Brazil. The manuscript is very well written and understandable. The methods are adequately explained, although some minor details can be added to improve reproducibility.*

In infectious disease modelling, fitting disease data to a known statistical distribution is a common first step of the analyses, but oftentimes it is overlooked. The first great contribution of this work to VL modellers is summarised in table 2 – the fitted distributions for country and state-level surveillance datasets. I do not recall seeing the gamma distribution in other VL studies, which usually apply the Poisson distribution. This has the potential to become an important reference for VL modelling exercises in Brazil.

The second main contribution is the effect of spatial scale and the importance of choosing the right dataset for your research question. This has been explored before in theory, but to the best of my knowledge this is the first time I see these scale comparisons in VL data from Brazil.

I recommend the acceptance of the manuscript after some minor details are clarified, specifically:

L125: Explain why the Federal District was not included in the dataset.

> This study largely aimed to compare distributional fits at spatial resolutions, using incidence by municipality and by state. Because the Federal District is not

a state with multiple municipalities, Visceral Leishmaniasis incidence cannot be aggregated to allow municipality-resolution and state-resolution incidence to be calculated. This clarification has been added to the text on line 133 of the revised manuscript.

L131: Give further info on exclusion criteria for municipalities. Were they excluded due to missing population data only or disease data as well?

Excluded municipalities were not present in the IBGE population data. This sentence was rewritten to make this clearer, and appears on line 140 of the revised manuscript.

L222: It would be very relevant, for reproducibility, if the R code used in the analyses were uploaded as supporting information or uploaded to a code repository such as GitHub.

The third supplemental material, originally containing the original data, has been replaced with S3_File.zip. This contains a folder with the original incidence data as well as the R code used for these analyses.

L289: "different processes driving disease occurrence" – this made me think about the differences between disease occurrence and disease notification, which is the actual data that was analysed. Your discussion might also include comments on how underreporting of VL affect these analyses. It is very likely that underreporting varies among Brazilian states.

This is a very important note. This fact is very present in infectious disease literature. A paragraph has been added to the discussion highlighting that this study uses reported cases, which represent a subset of the true number of cases. This paragraph begins on line 418 of the revised document.

Fig. 1: In the map of Brazilian States there are some extra lines appearing where they should not, more specifically inside the states MS, GO, SP, ES and PE. This is probably an artefact of the shapefile that was used, it should be corrected.

We appreciate catching this detail. This has been fixed.

***Reviewer #2**: I thank the authors for this work on an interesting subject. I suggest minor revision according to these comments:*
Introduction section:
1. "Urban area" Lines 98 and 101: Please check !

The citations associated with the statements indicate that Visceral Leishmaniasis has a canine reservoir in Brazil and that the disease has been seen in urban settings and is associated with urbanization in recent decades, in contrast to having been seen in rural settings previously. The wording has changed to

reflect that canine reservoirs are not exclusively in urban settings and that the recent shift to greater incidence in urban settings occurred since 1980 rather than the most recent years.

2. Authors don't precise Leishmania species caused VL in Brazil.

Cases of VL in Brazil have been attributed to *L. infantum*. This has been added on line 90 of the revised manuscript.

MM section:
1. Data collection: Please add subtitle for the first paragraph

This has been added.

2. 22 Brazilian states were included; please add data about epidemiological status: endemic/non endemic state!

Classifications of states as endemic/non endemic according to the Brazilian Ministries of Health, with citations, have been added, starting on line 104 of the revised manuscript as well as in Table 2.

Discussion section:
Authors must discuss according to other parameters: epidemiological status of the study area, type of infection....

This point of epidemiological status has been included. Similarly, other heterogeneities across Brazil are not accounted for in the distribution fitting process, but should be considered in any modeling studies that follow. This has been added in the paragraph starting on line 409 of the revised manuscript as well as on lines 424-428.

The point of the type of infection connects with the other reviewer's comment regarding underreporting of cases, as the most severe cases are less commonly underreported. The severity of infection has been added as a point describing underreporting on lines 446-448 of the revised manuscript.

In addition to these specific comments, the manuscript has been checked for any spelling/grammatical errors as well as citations and other formatting matters. We appreciate your consideration of our revised manuscript and hope to hear from you soon.

Thank you,

Joseph L Servadio
