# Supplementary material for: Information differences across spatial resolutions and scales for disease surveillance and analysis: The case of Visceral Leishmaniasis in Brazil
Source: PLoS One. 2020 Jul 17;15(7):e0235920. doi: 10.1371/journal.pone.0235920 (PMC7367469; doi:10.1371/journal.pone.0235920)
Supplement: Attachment — Submitted filename: PONE-D-20-06883_R1_reviewer.pdf [file pone.0235920.s005.pdf]

PLOS ONE

Information differences across spatial resolutions and scales for disease surveillance and analysis: The case of Visceral Leishmaniasis in Brazil

--Manuscript Draft--

Manuscript Number:	PONE-D-20-06883R1
Article Type:	Research Article
Full Title:	Information differences across spatial resolutions and scales for disease surveillance and analysis: The case of Visceral Leishmaniasis in Brazil
Short Title:	Sensitivity to spatial resolution and scale for infectious disease surveillance
Corresponding Author:	Joseph L Servadio, ScM<br>University of Minnesota School of Public Health<br>Minneapolis, MN UNITED STATES
Keywords:	visceral leishmaniasis; surveillance; scale; resolution; distributions; KL divergence
Abstract:	Nationwide disease surveillance at a high spatial resolution is desired for many infectious diseases, including Visceral Leishmaniasis. Statistical and mathematical models using data collected from surveillance activities often use a spatial resolution and scale either constrained by data availability or chosen arbitrarily. Sensitivity of model results to the choice of spatial resolution and scale is not, however, frequently evaluated. This study aims to determine if the choice of spatial resolution and scale are likely to impact statistical and mathematical analyses. Visceral Leishmaniasis in Brazil is used as a case study. Probabilistic characteristics of disease incidence, representing a likely outcome in a model, are compared across spatial resolutions and scales. Best fitting distributions were fit to annual incidence from 2004 to 2014 by municipality and by state. Best fits were defined as the distribution family and parameterization minimizing the sum of absolute error, evaluated through a simulated annealing algorithm. Gamma and Poisson distributions provided best fits for incidence, both among individual states and nationwide. Comparisons of distributions using Kullback-Leibler divergence shows that incidence by state and by municipality do not follow distributions that provide equivalent information. Few states with Gamma distributed incidence follow a distribution closely resembling that for national incidence. These results demonstrate empirically how choice of spatial resolution and scale can impact mathematical and statistical models.
Order of Authors:	Joseph L Servadio, ScM
	Gustavo Machado
	Julio Alvarez
	Francisco Edilson de Ferreira Lima Junior
	Renato Vieira Alves
	Matteo Convertino
Response to Reviewers:	The reviewer comments are seen in quotes, and responses to specific comments are found directly below relevant comments, with each paragraph beginning with the lead author's initials, JLS.<br><br>"Reviewer #1: This is a very relevant study that addresses the effect of spatial scales on visceral leishmaniasis surveillance data from Brazil. The manuscript is very well written and understandable. The methods are adequately explained, although some minor details can be added to improve reproducibility.<br><br>In infectious disease modelling, fitting disease data to a known statistical distribution is a common first step of the analyses, but oftentimes it is overlooked. The first great contribution of this work to VL modellers is summarised in table 2 – the fitted distributions for country and state-level surveillance datasets. I do not recall seeing the gamma distribution in other VL studies, which usually apply the Poisson distribution.

Powered by Editorial Manager® and ProduXion Manager® from Aries Systems Corporation

This has the potential to become an important reference for VL modelling exercises in Brazil.

The second main contribution is the effect of spatial scale and the importance of choosing the right dataset for your research question. This has been explored before in theory, but to the best of my knowledge this is the first time I see these scale comparisons in VL data from Brazil.

I recommend the acceptance of the manuscript after some minor details are clarified, specifically:

L125: Explain why the Federal District was not included in the dataset."

JLS: This study largely aimed to compare distributional fits at spatial resolutions, using incidence by municipality and by state. Because the Federal District is not a state with multiple municipalities, Visceral Leishmaniasis incidence cannot be aggregated to allow municipality-resolution and state-resolution incidence to be calculated. This clarification has been added to the text on line 133 of the revised manuscript.

"L131: Give further info on exclusion criteria for municipalities. Were they excluded due to missing population data only or disease data as well?"

JLS: Excluded municipalities were not present in the IBGE population data. This sentence was rewritten to make this clearer, and appears on line 140 of the revised manuscript.

"L222: It would be very relevant, for reproducibility, if the R code used in the analyses were uploaded as supporting information or uploaded to a code repository such as GitHub."

JLS: The third supplemental material, originally containing the original data, has been replaced with S3_File.zip. This contains a folder with the original incidence data as well as the R code used for these analyses.

"L289: “different processes driving disease occurrence” – this made me think about the differences between disease occurrence and disease notification, which is the actual data that was analysed. Your discussion might also include comments on how underreporting of VL affect these analyses. It is very likely that underreporting varies among Brazilian states."

JLS: This is a very important note. This fact is very present in infectious disease literature. A paragraph has been added to the discussion highlighting that this study uses reported cases, which represent a subset of the true number of cases. This paragraph begins on line 418 of the revised document.

"Fig. 1: In the map of Brazilian States there are some extra lines appearing where they should not, more specifically inside the states MS, GO, SP, ES and PE. This is probably an artefact of the shapefile that was used, it should be corrected."

JLS: We appreciate catching this detail. This has been fixed.

"Reviewer #2: I thank the authors for this work on an interesting subject. I suggest minor revision according to these comments:
Introduction section:
1. “Urban area” Lines 98 and 101: Please check !"

JLS: The citations associated with the statements indicate that Visceral Leishmaniasis has a canine reservoir in Brazil and that the disease has been seen in urban settings and is associated with urbanization in recent decades, in contrast to having been seen in rural settings previously. The wording has changed to reflect that canine reservoirs are not exclusively in urban settings and that the recent shift to greater incidence in urban settings occurred since 1980 rather than the most recent years.

"2. Authors don't precise Leishmania species caused VL in Brazil."

Powered by Editorial Manager® and ProduXion Manager® from Aries Systems Corporation

	JLS: Cases of VL in Brazil have been attributed to L. infantum. This has been added on line 90 of the revised manuscript.<br><br>"MM section:<br>1. Data collection: Please add subtitle for the first paragraph"<br><br>JLS: This has been added.<br><br>"2. 22 Brazilian states were included; please add data about epidemiological status: endemic/non endemic state!"<br><br>JLS: Classifications of states as endemic/non endemic according to the Brazilian Ministries of Health, with citations, have been added, starting on line 104 of the revised manuscript as well as in Table 2.<br><br>"Discussion section:<br>Authors must discuss according to other parameters: epidemiological status of the study area, type of infection...."<br><br>JLS: This point of epidemiological status has been included. Similarly, other heterogeneities across Brazil are not accounted for in the distribution fitting process, but should be considered in any modeling studies that follow. This has been added in the paragraph starting on line 409 of the revised manuscript as well as on lines 424-428.<br><br>JLS: The point of the type of infection connects with the other reviewer's comment regarding underreporting of cases, as the most severe cases are less commonly underreported. The severity of infection has been added as a point describing underreporting on lines 446-448 of the revised manuscript.
**Additional Information:**	
**Question**	**Response**
**Financial Disclosure**<br><br>Enter a financial disclosure statement that describes the sources of funding for the work included in this submission. Review the submission guidelines for detailed requirements. View published research articles from *PLOS ONE* for specific examples.<br><br>This statement is required for submission and **will appear in the published article** if the submission is accepted. Please make sure it is accurate.	This work was funded by the Academic Health Center Faculty Research Development Grant Program (FRD no. 16.36) from the University of Minnesota Twin-Cities, received by JA and MC. The funders played no role in study design, data collection and analysis, decision to publish, or preparation of the manuscript.

**Unfunded studies**<br>Enter: *The author(s) received no specific funding for this work.*<br><br>**Funded studies**<br>Enter a statement with the following details:<br>• Initials of the authors who received each award<br>• Grant numbers awarded to each author<br>• The full name of each funder<br>• URL of each funder website<br>• Did the sponsors or funders play any role in the study design, data collection and analysis, decision to publish, or preparation of the manuscript?<br>• **NO** - Include this sentence at the end of your statement: *The funders had no role in study design, data collection and analysis, decision to publish, or preparation of the manuscript.*<br>• **YES** - Specify the role(s) played.<br><br>* typeset	
**Competing Interests**<br><br>Use the instructions below to enter a competing interest statement for this submission. On behalf of all authors, disclose any competing interests that could be perceived to bias this work—acknowledging all financial support and any other relevant financial or non-financial competing interests.<br><br>This statement **will appear in the published article** if the submission is accepted. Please make sure it is accurate. View published research articles from *PLOS ONE* for specific examples.	The authors have declared that no competing interests exist.

Powered by Editorial Manager® and ProduXion Manager® from Aries Systems Corporation

**NO authors have competing interests**<br><br>Enter: *The authors have declared that no competing interests exist.*<br><br>**Authors with competing interests**<br><br>Enter competing interest details beginning with this statement:<br><br>*I have read the journal's policy and the authors of this manuscript have the following competing interests: [insert competing interests here]*<br><br>* typeset	
**Ethics Statement**<br><br>Enter an ethics statement for this submission. This statement is required if the study involved:<br><br>• Human participants<br>• Human specimens or tissue<br>• Vertebrate animals or cephalopods<br>• Vertebrate embryos or tissues<br>• Field research<br><br>Write "N/A" if the submission does not require an ethics statement.<br><br>General guidance is provided below. Consult the submission guidelines for detailed instructions. **Make sure that all information entered here is included in the Methods section of the manuscript.**	N/A

Powered by Editorial Manager® and ProduXion Manager® from Aries Systems Corporation

**Format for specific study types**<br><br>**Human Subject Research (involving human participants and/or tissue)**<br>• Give the name of the institutional review board or ethics committee that approved the study<br>• Include the approval number and/or a statement indicating approval of this research<br>• Indicate the form of consent obtained (written/oral) or the reason that consent was not obtained (e.g. the data were analyzed anonymously)<br><br>**Animal Research (involving vertebrate animals, embryos or tissues)**<br>• Provide the name of the Institutional Animal Care and Use Committee (IACUC) or other relevant ethics board that reviewed the study protocol, and indicate whether they approved this research or granted a formal waiver of ethical approval<br>• Include an approval number if one was obtained<br>• If the study involved *non-human primates*, add *additional details* about animal welfare and steps taken to ameliorate suffering<br>• If anesthesia, euthanasia, or any kind of animal sacrifice is part of the study, include briefly which substances and/or methods were applied<br><br>**Field Research**<br><br>Include the following details if this study involves the collection of plant, animal, or other materials from a natural setting:<br>• Field permit number<br>• Name of the institution or relevant body that granted permission	
**Data Availability**<br><br>Authors are required to make all data underlying the findings described fully available, without restriction, and from the time of publication. PLOS allows rare exceptions to address legal and ethical concerns. See the PLOS Data Policy and FAQ for detailed information.	Yes - all data are fully available without restriction

A Data Availability Statement describing where the data can be found is required at submission. Your answers to this question constitute the Data Availability Statement and **will be published in the article**, if accepted.<br><br>**Important:** Stating 'data available on request from the author' is not sufficient. If your data are only available upon request, select 'No' for the first question and explain your exceptional situation in the text box.<br><br>Do the authors confirm that all data underlying the findings described in their manuscript are fully available without restriction?	
**Describe where the data may be found in full sentences. If you are copying our sample text, replace any instances of XXX with the appropriate details.**<br><br>• If the data are **held or will be held in a public repository**, include URLs, accession numbers or DOIs. If this information will only be available after acceptance, indicate this by ticking the box below. For example: *All XXX files are available from the XXX database (accession number(s) XXX, XXX.).*<br>• If the data are all contained **within the manuscript and/or Supporting Information files**, enter the following: *All relevant data are within the manuscript and its Supporting Information files.*<br>• If neither of these applies but you are able to provide **details of access elsewhere**, with or without limitations, please do so. For example:<br><br>*Data cannot be shared publicly because of [XXX]. Data are available from the XXX Institutional Data Access / Ethics Committee (contact via XXX) for researchers who meet the criteria for access to confidential data.*<br><br>*The data underlying the results presented in the study are available from (include the name of the third party*	All relevant data are within the manuscript and its Supporting Information files. They can be found in S3_File.zip.

Powered by Editorial Manager® and ProduXion Manager® from Aries Systems Corporation

and contact information or URL).<br>• This text is appropriate if the data are owned by a third party and authors do not have permission to share the data.<br><br>* typeset	
Additional data availability information:	

Manuscript

Click here to access/download;Manuscript;Servadio_VL_PlosOne_Apr2020_R

Information differences across spatial resolutions and scales for disease surveillance and analysis: The case of Visceral Leishmaniasis in Brazil

Short title: Sensitivity to spatial resolution and scale for infectious disease surveillance

Joseph L Servadio,[1]* Gustavo Machado,[2] Julio Alvarez,[3, 4] Francisco Edilson de Ferreira Lima Júnior,[5] Renato Vieira Alves,[5] Matteo Convertino[6]

[1] Division of Environmental Health Sciences, University of Minnesota School of Public Health, 420 Delaware Street SE, Minneapolis, MN, USA.

[2] Department of Population Health and Pathobiology, College of Veterinary Medicine, North Carolina State University, 1060 William Moore Drive, Raleigh, NC, USA.

[3] VISAVET Health Surveillance Center, Universidad Complutense, Avda Puerta de Hierro S/N, 28040 Madrid, Spain.

[4] Departamento de Sanidad Animal, Facultad de Veterinaria, Universidad Complutense, Avda Puerta de Hierro S/N, 28040 Madrid, Spain

[5] Secretaria de Vigilância em Saúde, Ministério da Saúde (SVS-MH), Brasília, Brazil

[6] Nexus Group, Graduate School of Information Science and Technology and GI-CoRE Station for Big-Data and Cybersecurity, Hokkaido University, Sapporo, Hokkaido, Japan.

* Corresponding author: Joseph L Servadio. Division of Environmental Health Sciences, University of Minnesota School of Public Health, MMC807, 420 Delaware Street SE, Minneapolis, MN, 55455, USA.

serva024@umn.edu

Abstract

Nationwide disease surveillance at a high spatial resolution is desired for many infectious diseases, including Visceral Leishmaniasis. Statistical and mathematical models using data collected from surveillance activities often use a spatial resolution and scale either constrained by data availability or chosen arbitrarily. Sensitivity of model results to the choice of spatial resolution and scale is not, however, frequently evaluated. This study aims to determine if the choice of spatial resolution and scale are likely to impact statistical and mathematical analyses. Visceral Leishmaniasis in Brazil is used as a case study. Probabilistic characteristics of disease incidence, representing a likely outcome in a model, are compared across spatial resolutions and scales. Best fitting distributions were fit to annual incidence from 2004 to 2014 by municipality and by state. Best fits were defined as the distribution family and parameterization minimizing the sum of absolute error, evaluated through a simulated annealing algorithm. Gamma and Poisson distributions provided best fits for incidence, both among individual states and nationwide. Comparisons of distributions using Kullback-Leibler divergence shows that incidence by state and by municipality do not follow distributions that provide equivalent information. Few states with Gamma distributed incidence follow a distribution closely resembling that for national incidence. These results demonstrate empirically how choice of spatial resolution and scale can impact mathematical and statistical models.

Keywords: Visceral Leishmaniasis; surveillance; scale; resolution; distributions; KL divergence

1. Introduction

Infectious disease research often relies on data generated through passive or active surveillance activities, which can suffer from important limitations due to variation in methods and capacities for data collection [1, 2]. Typically, researchers aim to collect data at a high spatial resolution, that is, in the form of small surveillance units such as counties or municipalities rather than states or nations [3, 4], though this may not always be seen as beneficial [5]. Conducting surveillance at a high spatial resolution, however, is often unrealistic when considering large areas and constrained resources [6, 7].

Data collected from infectious disease surveillance activities are often used in researching involving mathematical or statistical models. In such analyses, matters related to data quality are of concern. The choice of spatial resolution is often based on data availability or chosen arbitrarily, with little attention given to whether this decision may impact model results. Aggregating data into larger spatial units can aid in computational efficiency, but creates the risk of introducing ecological fallacy [8] and masking heterogeneity within those larger units if conclusions are drawn inappropriately [9–11]. This would be particularly problematic when aiming to seek disease etiologies. Previous studies using mathematical or statistical models have investigated the importance of high resolution data by repeating analyses using data at different resolutions and then comparing results [10–12].

An additional challenge to high quality surveillance is the need for surveillance over a large spatial scale, referring to the entire area where surveillance is being conducted. Here, spatial scale differs from spatial resolution in their definitions as follows: spatial scale refers to the total spatial area being examined, while spatial resolution refers to the size of the individual spatial units within that area. Large-scale surveillance can be particularly challenging for nations with large land areas and populations. In these circumstances, there is potential benefit in identifying a smaller area, such as a state or group of states, where surveillance can adequately estimate the national disease burden. The characteristic of having smaller

areas representative of the whole for a large range of sizes is known as scale invariance or fractality [13]. Scale invariance is ubiquitous in many socio-ecological patterns such as finance [14], ecology [15], biochemical processes [16], and biology across time scales [17].

Scale invariance is an infrequently examined concept in infectious disease surveillance and epidemiology in general, though it has relevance to many forms of data analysis or modeling. Scale invariance in infectious disease research is more frequently used to describe scale-free networks, typically applied to human communicable diseases [18] or transmission paths of infectious diseases [19]. For practical purposes in epidemiology, identifying smaller regions that represent a larger area or even an entire nation could allow the design of targeted surveillance strategies and conserve resources [20]. Even in the absence of true scale invariance, self-similarity can be observed [13] where some smaller areas can be used to describe the whole. In other situations, however, the spatial scale of interest impacted the physical processes being studied [21].

The topics of spatial resolution and scale are relevant for research pertaining to numerous health outcomes. Here, Visceral Leishmaniasis is examined as a case study. Visceral Leishmaniasis (VL), caused by a *Leishmania infantum* parasite (known in Latin America as *Leishmania chagasi*) [22], is the most severe form of Leishmaniasis [23] and is fatal in the vast majority of untreated cases [24]. The parasite is typically transmitted from an infected to a non-infected host through the bites of phlebotomine sand flies [25]. Visceral Leishmaniasis presentation can include symptoms such as fever, enlargement of the spleen and liver, and anaemia [23]. Worldwide, there are between 50,000 and 90,000 estimated VL cases yearly [23].

Brazil is one of ten world nations with the greatest VL burdens [23], with the remaining nations located primarily in East Africa and South and Southeast Asia [23]. Estimates report that 90% of VL cases that occur in the Americas occur in Brazil [23] where, canines are a disease reservoir [24, 26, 27]. Areas of

Brazil that previously had accounted for only 15% of all cases reported nationally now can see nearly half of the nation's cases [27]. The disease has also become more common in urban areas in recent decades [26, 28, 29], making it a major public health concern and an important target for surveillance programs. As of 2015, based on the data used for this study, the Federal District and 18 of the 26 states in Brazil meet the criterion of being an endemic state for VL, which is seeing at least one case in all three previous years [30]. The states that were not endemic at the time are Acre, Amapá, Amazonas, Espírito Santo, Paraná, Rondônia, Roraima, and Santa Catarina [30, 31]. As of 2019, Espírito Santo and Paraná became endemic states [31].

This study aims to assess the potential impact of using different spatial resolutions and scales on statistical and mathematical models using surveillance data applied to VL cases in Brazil. In order to do so, two objectives are pursued: (1) to determine if surveillance using incidence by state or municipality leads to different conclusions regarding disease distribution; and (2) to determine if conducting VL surveillance on a region within Brazil would adequately characterize the nation's VL incidence. This is done by using best fitting probability distributions to describe disease data without incorporating outside information. A conceptual visualization of the study aims are presented in Figure 1. Prior to conducting statistical analyses or models, researchers may need to decide whether to consider data using different spatial resolutions as well as the scale of analysis; the results of this study will provide insight into whether the subsequent results may be sensitive to this decision.

Fig 1. Graphical overview of the study objectives: (a) fit distribution to annual incidence of Visceral Leishmaniasis (VL) by state, (b) fit distribution to annual incidence of VL by municipality, (c) fit distributions to annual incidence of VL by municipality within each state. Comparisons of these fitted distributions indicate whether characterizing VL incidence by state or municipality are equivalent, impacting statistical analyses using these data.

2. Methods and Materials

2.1. Study setting and data

The setting for this study is Brazil, the largest nation in South America in both land area and population. Case data were provided by the Brazilian Ministries of Health [32] and include VL case counts by municipality nationwide, with the exception of the Federal District, totaling 26 states and 5,561 municipalities. The Federal District was excluded because it is not a state with multiple municipalities, and therefore cannot be aggregated to differentiate between the municipality resolution and the state resolution. Yearly case counts by municipality between 2004 and 2014 are reported. Annual populations for each municipality are publicly available through the Brazilian Institute of Geography and Statistics [33] to calculate annual incidence, discretized to represent cases per 100,000 population per year. Population data were available for all years except 2007 and 2010. In these two years, the arithmetic means of the populations of the two adjacent years were used in place of the missing populations. Population data were available for 5,538 of the municipalities with VL data, providing the final sample for this study.

2.2 Inferring probability distributions

This study compares spatial resolutions and scales using probability distributions rather than by fitting models with assumptions and conducting a sensitivity analysis. Best fitting distributions from multiple considered distributional families were fit for (1) annual incidence for each individual state using the municipality as the unit of surveillance; (2) annual incidence nationwide using the municipality as the unit of surveillance; and (3) annual incidence nationwide using the state as the unit of surveillance (Figure 1). All 11 years of observation were included.

Common candidate distributions were selected based on exploratory analyses and suitability for discrete incidence data, and wide ranges of parameters for each distribution were tested. The Poisson, Zero Inflated Poisson (ZIP), and Zero One Inflated Poisson (ZOIP) [34] distributions were selected as candidate distributions along with the Gamma, Exponential, Power Law, and Uniform distributions rounded to fit discrete data. These are described in Table 1.

Table 1. Candidate distributions used for fitting distributions

Distribution	Probability mass function
Poisson	$P(X = x) = \frac{\theta^x}{x!} \exp(-\theta)$
Zero Inflated Poisson (ZIP)	$P(X = 0) = \alpha + (1 - \alpha) \exp(-\theta)$<br>$P(X = x) = (1 - \alpha) \frac{\theta^x}{x!} \exp(-\theta), x > 1$
Zero One Inflated Poisson (ZOIP)	$P(X = 0) = \alpha + (1 - \alpha - \beta) \exp(-\theta)$<br>$P(X = 1) = \beta + (1 - \alpha - \beta)\, \theta \exp(-\theta)$<br>$P(X = x) = (1 - \alpha - \beta) \frac{\theta^x}{x!} \exp(-\theta), x > 1$
Rounded Exponential	$P(X = x) = \int_0^{x+0.5} f(y)\, dy - \int_0^{x-0.5} f(y)\, dy$<br>$f(y) = \theta \exp(-\theta\, y)$
Rounded Gamma	$P(X = x) = \int_0^{x+0.5} f(y)\, dy - \int_0^{x-0.5} f(y)\, dy$<br>$f(y) = \frac{\beta^\alpha}{\Gamma(\alpha)} y^{\alpha-1} \exp(-\beta\, y)$
Rounded Power Law	$P(X = x) = \int_0^{x+0.5} f(y)\, dy - \int_0^{x-0.5} f(y)\, dy$<br>$f(y) = \frac{\alpha - 1}{x_{min}} \left(\frac{x}{x_{min}}\right)^{-\alpha}, x > x_{min}$
Rounded Uniform	$P(X = x) = \frac{1}{b - a}, x \in \mathbb{Z} \cap [a, b]$

Each distribution was evaluated for the optimal parameter set that minimizes the sum of absolute error (SAE), defined as

$SAE = \sum_x |p(x) - p_{obs}(x)|$ (Equation 1)

where $p(x)$ represents the probability of observing an incidence of x cases per 100,000 person-years based on the proposed distribution indicated in Table 1 and $p_{obs}(x)$ represents the observed proportion of incidence values equaling x. This measure compares similarities between the proposed distributions and observed data and is less sensitive to outliers than other measures [35].

The optimal parameter set for each distribution was found through simulated annealing [36], an optimization algorithm based on Monte Carlo sampling. The algorithm was run with three chains for 50,000 simulations to assure convergence. Convergence was reached if each of the three chains produced identical parameter values, indicating a lack of movement to other parameter values, for at least 500 iterations, as well as one of the following hierarchical criteria: (1) final parameter values across pairs of chains had an absolute difference of less than 0.01; (2) final parameter values had an absolute difference of less than 0.1 and associated SAE values had an absolute difference of less than 0.01; (3) SAE values had an absolute difference of less than 0.001. The second and third criteria were necessary due to some parameterizations having very similar SAE values. If convergence was not reached in 50,000 iterations, the three chains were restarted with the parameterization that led to the lowest SAE value in the chain as initial values, and the simulated annealing algorithm was repeated, increasing the simulation count by 5,000. This was repeated until convergence was reached.

2.3 Comparing Distributions

2.3.1 Sensitivity to spatial resolution

The first aim of comparing distributions is to determine if different inferential conclusions may be reached using the municipality or the state as the resolution of nationwide surveillance. This was done by comparing the fitted state-resolution distribution to an expected state-resolution distribution for the nation based on the fitted municipality-resolution distribution for the nation. This expected distribution was

generated empirically by drawing Monte Carlo samples from the fitted municipality-resolution distribution.

If incidence is denoted by X as a random variable following the fitted municipality-resolution distribution for the nation; states are denoted by s; state s has n_s municipalities, denoted by m; and municipality m has a population of p_{my} in year y, this empirical distribution was generated by drawing 1,000 samples of

$$\frac{\sum_{m=1}^{s} p_{my} x_m}{\sum_{m=1}^{s} p_{my}} \quad \text{(Equation 2)}$$

for each state under each year of observation. The numerator of Equation 2 draws values of X_m for each municipality in a state and multiplies the value by the population of the municipality to sample a case count for the municipality. The sum of these is divided by the total state population to produce an expected state-resolution incidence for a year based on the municipality-resolution distribution for incidence.

Relative proportions of incidence values in these simulated values were compared to the probabilities of each incidence value from the fitted state-resolution distribution through Kullback-Leibler (KL) divergence [37]. KL divergence represents the additional information needed when using one distribution to describe data from another distribution. By measuring dissimilarity, it has an inverse relationship to Mutual Information, which represents similarity of variables [37]. Thus, KL divergence is a measure of the Value of Information [38]. For two random variables, denoted A and B, the KL divergence from A to B, compared to the Shannon entropy in the distribution of A, shows a relative increase in information, using bits as units, needed to describe the distribution of B with that of A [37]. This is shown in the ratio of KL divergence to Shannon entropy (denoted *H),* which can be defined as the Required Relative Information Gain (RRIG), where

$$RRIG = \frac{KL_{A \to B}}{H_A}$$ (Equation 3)

shows the needed increase in information for the distribution of B to describe data from the distribution of A [37]. A value of 1 represents an information increase by 100%, or a doubling of information, though this is not an upper bound. A large RRIG value is indicative of distinct differences between distributions, indicating that characterizations of VL incidence are sensitive to the resolution of surveillance. An RRIG above 5% was selected as a threshold for having a distinct difference in distribution.

2.3.2 Sensitivity to spatial scale

The aim of comparing spatial scales involves comparing the municipality-resolution distributions of each state and of the nation. In the presence of scale invariance, individual states would have the same or similar distributions, which would be similar to the distribution for the nation. Distributions for municipality-resolution case counts for the nation and each state were compared through RRIG.

All analyses were performed using R version 3.6.0 [39]. The 'poweRlaw' package was used to calculate probability density and mass for the Power Law distribution [40].

3. Results

Of the 26 states in Brazil, 22 were included in analyses since they all observed more than five nonzero unique annual municipality-resolution incidence values over the study period (Table S1). The remaining four states were excluded because their incidences over the 11 years did not provide enough unique values to reliably fit a distribution. All 18 endemic states from 2015 [31] were included as well as Espírito

Santo, Paraná, Rondônia, and Roraima. Figure 2 (a and b) shows total case counts by state and by municipality over the entire time period.

Fig 2. Total case counts by (a) municipality and (b) state between 2004 and 2014

3.1 Fitted distributions

The uniform distribution for nationwide municipality-resolution incidence was not able to converge after increasing the iteration count to 200,000. All other distributions converged to optimal values. The best fitting municipality-resolution distributions for individual states varied. Annual incidence values from ten states were best fit by the rounded Gamma distribution, incidences from seven states were best fit by the Poisson distribution, incidences from three states were best fit by the Zero Inflated Poisson distribution, and incidences from two states were best fit by the Zero One Inflated Poisson distribution. Specific parameters are shown by state in Table 2. Plots of the probability mass functions of each state's municipality-resolution distribution are shown in Figure 3. Nationwide, the best fitting distribution for municipality-resolution incidence was the Gamma distribution, and the best fitting distribution for state-resolution incidence was the Zero One Inflated Poisson distribution (Table 2). No notable differences were seen in distributional fit among VL endemic and non-endemic states.

Table 2. Distributions for municipality-resolution incidences by state, municipality-resolution incidence nationwide, and state-resolution incidence nationwide. KL/H calculated from Equation 3 shows comparisons of fitted distributions to that for the nationwide municipality-resolution distribution (reference).

State	Distribution	Mean	Variance	KL/H
AL*	Poisson(0.234)	0.234	0.234	0.456

BA*	Gamma(0.078, 0.022)	3.545	161.157	0.191
CE*	Gamma(0.191, 0.038)	5.026	132.272	0.824
ES	Gamma(0.003, 0.002)	1.500	750.000	0.084
GO*	Gamma(0.016, 0.016)	1	62.5	0.009
MA*	ZOIP(0.599, 0.004, 7.292)	2.899	15.605	1.154
MG*	Gamma(0.024, 0.020)	1.2	60	0.001
MS*	Poisson(0.378)	0.378	0.378	0.791
MT*	Gamma(0.015, 0.003)	5	1666.667	0.007
PA*	Gamma(0.094, 0.022)	4.273	194.215	0.289
PB*	Poisson(0.077)	0.077	0.077	0.155
PE*	ZIP(0.819, 4.193)	0.759	3.365	0.263
PI*	ZIP(0.723, 14.44)	4.000	45.759	0.878
PR	Poisson(0.003)	0.003	0.003	0.151
RJ*	Gamma(0.002, 0.009)	0.222	24.691	0.108
RN*	Poisson(0.085)	0.085	0.085	0.165
RO	Poisson(0.013)	0.013	0.013	0.128
RR	Gamma(0.015, 0)	15	15000	0.021
RS*	ZIP(0.998, 4.338)	0.009	0.046	0.146
SE*	ZOIP(0.776, 0.006, 5.254)	1.151	5.43	0.388
SP*	Gamma(0.014, 0.013)	1.077	82.840	0.01
TO*	Poisson(0.450)	0.450	0.450	0.960
Brazil (Municipality)	Gamma(0.024, 0.017)	1.411	83.045	Reference
Brazil (State)	ZOIP(0.379, 0.074, 2.82)	1.617	3.353	0.425

* denotes states considered endemic for Visceral Leishmaniasis in 2015 [30, 31]

Fig 3. Probability mass functions for distributions fit to municipality-resolution incidences among states and for the nation. Incidences were fit by either (a) a Poisson distribution, including distributions with zero and one inflations, or (b) a Gamma distribution.

3.2 Comparisons across resolutions and scales

The RRIG from Equation 3 was used to quantify the similarities between distributions. The distributions fitted to state-resolution incidence and to the empirical distribution created from the national municipality-resolution distribution and Equation 2 were first compared to determine the sensitivity to the resolution of surveillance data. The RRIG was 0.425, representing a needed increase of information by 42.5% (Table 2). These results are indicative of strong sensitivity to the resolution of surveillance; the distribution for VL incidence by state does not accurately describe the distribution of incidence by municipality.

Comparisons between individual states' municipality-resolution incidence and national municipality-resolution incidence are shown in Table 2 using RRIG from Equation 3. The nationwide, municipality-resolution distribution is used as the reference for comparisons. The results show that six of the 22 states had incidence following a distribution close to that of the nation (RRIG<0.05) (Table 2, Figure 4). Any of these states could individually describe municipality-resolution incidence of the nation. Because not all states adequately characterize national burden, true scale invariance was not seen, though self-similarity was seen in the selected states. The states that exhibited some self-similar behaviors all followed a Gamma distribution with generally similar parameters, particularly low values of shape parameters. Many of these states were located near the center of the nation (Figure 4).

Fig 4. Expected values and families of fitted municipality-resolution distributions. States outlined in red had low KL divergence to Shannon entropy ratio with the national municipality-resolution distribution, indicating self-similarity.

4. Discussion

This study aimed to assess the importance of the spatial scale and resolution used for VL surveillance and subsequent quantitative analyses. This is also reflective of the dynamics of VL at different scales determined by the distributions of incidence. Probability distributions were fit to incidences at different spatial resolutions and scales and then compared to determine if distributional fit was sensitive to the choice of scale and resolution. Aggregating municipality-resolution incidences into state-resolution incidences led to notably different probabilistic characteristics of disease burden, suggesting the existence of different processes driving disease occurrence at the two resolutions. When continuing surveillance at the municipality resolution, six states' incidences follow distributions that adequately describe those of each other as well as the nation of Brazil. While our results provide evidence against true invariance to resolution and scale, some self-similarity is seen in both distributional parameters and moments. This happens for states that are following a Gamma distribution, which implies medium-long range dispersal of cases and a potential tendency toward a power-law distribution for small scale and shape parameters.

The self-similarity seen in six states does not indicate that significant resources can be saved in Brazil by concentrating surveillance in a smaller area because they are not representative of the other states. The remaining states still need to undergo surveillance in order for their VL burden to be adequately characterized. Furthermore, it is of interest for public health to know where all VL cases occur in order to intervene in an outbreak. If greater self-similarity were seen, it would largely be of interest to researchers who could potentially generalize results of a smaller area to the nation of Brazil through conducting more intensive data collection for additional data in a smaller area. However, because scale invariance was not

seen and self-similarity was seen in a small number of states, it is unlikely that descriptions of VL burden in a smaller region of Brazil are generalizable to the entire nation. These considerations consider the current observed state, for instance in case of a widespread propagation of the disease in long range.

Differences in municipality-resolution distributions among states suggest that different factors may influence VL risk across states. Environmental factors shown to influence VL case risk include vector populations, canine cases, precipitation [41], proximity to wooded areas [42], land use, deforestation [43], temperature, and humidity [44]. The question of resolution dependence is targeting whether the elementary unit at which we look into VL dynamics makes a difference for reproducing the distributional representation of VL incidence at the scale of analysis. These natural phenomena related to VL burden may differ across spatial scales and resolutions, similarly to other physical phenomena [45]. Though vector populations and precipitation do not explicitly conform to local political boundaries, different regions of Brazil likely see differences in these risk factors through differences in managing socio-ecological factors. These facts and the finding that distributions across states differ (also varying when resolution varies) are important considerations when analyzing disease data. It would be advisable to analyze data for individual locations [41, 44, 46] or use random effects [32].

The results from this study do not necessarily suggest that one spatial resolution is more “correct” than another or favor a particular resolution for analysis. The resolution for statistical analyses should rely on the research question being posed and desired interpretation of results. However, the resolution dependence implies that, assuming accuracy and precision in assigning municipalities to observed cases, aggregating incidence to the state resolution likely introduces ecological fallacy. Thus, high resolution is likely beneficial to capture disease dynamics accurately. For high-resolution incidence, the most likely VL dynamics are represented by the Gamma distribution. These considerations should be always taken into account when collecting and analyzing data because they indicate that the choice of resolution will impact model results and their interpretation. Data characterizations and analyses at one resolution are not

interchangeable for characterizations and analyses at the other resolution. A related point to note is that diligent surveillance is important when conducted at a finer spatial resolution to ensure accuracy of municipalities that are matched to cases.

This study is, to the authors' knowledge, the first to examine VL incidence for sensitivity to scale and resolution of surveillance data by finding best-fitting distributions to characterize incidence. Other studies have analyzed the fractality of other diseases, such as cholera, and how that is important for a simple estimation of disease spread in term of geography and magnitude [47]. Similar distribution fitting processes are used in veterinary epidemiology [48], but less frequently in human disease epidemiology. This analysis is important for informing future disease burden by providing location-specific estimates of expected annual incidence.

The findings of this study can benefit surveillance, healthcare infrastructure, digital epidemiology, and public health research focused on disease ecology. Care for an individual VL patient in Brazil, including diagnosis, treatment, and medical care, is estimated to be approximately $500 (US) (plus an additional $1470 (US) for secondary prophylaxis among VL patients with HIV) and lasts between seven and 20 days [49]. This is a high individual healthcare cost: yet, designing optimal surveillance that allows public health practitioners to understand and prevent VL is an incredibly valuable task socially and economically. These results and methods (applicable to any disease) can optimize disease data analysis and surveillance for the reduction of the systemic disease burden.

Using only VL incidence data and not introducing other data sources provides focus on what would be the outcome variable of a typical statistical analysis independently of any other predictors that may be introduced. Refitting models at multiple resolutions or scales assumes that the outcome, in this instance VL incidence, follows the same distribution in each scale and/or resolution. For example, using a lognormal regression model with two resolutions assumes that incidence at both resolutions follows a

lognormal distribution, which may not be correct. When analyzing municipality-resolution cases, not all states have distributions in the same family, and distributions following the same family have different parameterizations because of the likely differential importance of the underlying socio-environmental drivers. The latter point further motivates the use of Bayesian hierarchical models or other models, for instance statistical physics and/or information theoretic models, which are able to handle the information of scale and resolution controlling factors.

We show that the information theoretic RRIG can determine the amount of information needed to describe the data using different resolutions or scales. It can be used as an information theoretic tool for scaling (downscaling or upscaling, depending on the purpose) epidemiological data considering their value and underlying distributions.

An additional point of novelty is the use of the ZOIP and Gamma distributions to characterize VL incidence. Both distributions are uncommonly used for infectious disease incidence, despite closely fitting observed data. The ZOIP distribution offers the advantage of specifically fitting high frequencies of counts of one, describing single spurious cases. The Gamma distribution is advantageous for placing high probability on low values. More specifically into the statistical physics of disease ecology, the Gamma distribution has similarities to heavy tail distributions (for small shape and scale parameters) and ZOIP represents Poisson distributions highlighting local/random and medium-range disease dynamics. The higher statistical complexity (e.g. related to the number of parameters) of ZOIP reflects the random Poissonian nature of the disease with other factors, while the lower complexity of the Gamma reflects its more simple nature.

These analyses do not consider dependence on temporal resolution and scale although time and space for stochastic processes relate to each other. The data in this study include yearly case counts; having smaller time units such as months would allow for such consideration Additionally, distributions are assumed to

remain constant over the 11 years of observation considering the very minor variations in the inferred distributions that lead to consider VL dynamics at stationary state. Increases have been seen in VL cases over time [50], though case counts between 2000 and 2014 have remained more consistent compared to previous decades [51, 52], indicating that these results are not likely to be sensitive to this assumption. Populations over this time period by municipality generally showed small changes. The mean change in population by municipality was an increase of approximately 11% between 2004 and 2014, and the middle 90% of changes were between a 12% decrease and a 41% increase [33]. These considerations motivate extensions of this study to define the relationship between space and time for scale dependent processes.

Another assumption made in this study is the ability to fit a single probability distribution for VL incidence for the entire nation of Brazil. Since not all of the included states are considered endemic for VL [31], fitting a single distribution for incidence nationwide assumes that the same distribution can represent incidence in both endemic and non-endemic states. However, this consideration should be considered in the quantitative analyses that would follow from the results of this study. Other heterogeneities across the nation, such as affluence, urbanization, or climate, which may impact VL incidence, similarly are not considered for distribution fitting but should be accounted for during subsequent analyses.

The results of this study rely on the data collected. VL case data were collected through passive surveillance and notification to the Ministries of Health. It is commonly known that reported cases of infectious diseases only represent a portion of the total cases [53–55], commonly representing the most severe cases. This limits the accuracy of the data, and therefore distribution fitting, by the ability to report cases as well as the potential heterogeneous severity of VL cases. It is also likely that across locations in Brazil, amounts of underreporting of cases differ. The results of this study rely on the assumption that reported cases provide an adequate representation of disease burden. Furthermore, inclusion of both

endemic and non-endemic states in the analyses may lead to the inclusion of case data representing both typical VL incidence as well as atypical VL incidence. This could potentially affect distribution fitting if underlying processes leading to typical and atypical incidence differ.

A limitation of this study is the reliance on the criterion for determining differences when comparing distributions and algorithm used for determining best fitting distribution families and parameters. There are numerous methods for performing both tasks, and different methods may lead to slightly different conclusions. The methods of this study do, however, use assumption-free criteria in order to generate the results. A sensitivity analysis was conducted to determine if the number of samples drawn to generate the empirical state-resolution distribution described in section 2.2.1 using Equation 2 might impact RRIG values, and it was found that using 1,000; 2,000; 5,000; and 10,000 samples did not yield distinct difference in RRIG values and no differences in interpretations and conclusions.

Another important note is that this study used surveillance units of different sizes, examining aggregation of municipalities of differing land areas and populations and comparisons among states with different areas and populations. This results from using administrative districts, and still remains useful by using the units recorded in infectious disease surveillance. However, diseases know no political boundaries; yet, an ecosystem-based discretization to define homogeneous high resolution units would be preferable for surveillance such as one based on Digital Elevation Models from which to derive physical ecosystem boundaries that are relevant to disease spread. This would also help the control of diseases to assign to different political entities. A related topic of research is the existence of spatial autocorrelation in the data. Values of Moran's I using municipality-resolution incidence nationwide showed strong evidence of spatial clustering. This implies that disease dynamics are non-local as already highlighted by differing fitted distributions across states, which is consistent with previous works [56, 57]. Subsequent analyses on VL in Brazil would benefit from the use of methods that account for spatial autocorrelation. For the purposes of distribution fitting, finding distributional families that most accurately characterize incidence

is of greater importance than determining a covariance structure that most accurately reflects autocorrelation. Determining clusters and covariance structures is an important component of analysis that follows the results of this study.

5. Conclusions

The choice of spatial resolution and scale in infectious disease research is shown to have an impact on results and conclusions of statistical and mathematical models. Finding sensitivity to the spatial resolution and spatial scale of VL surveillance data is of interest to both researchers and government officials for preparedness. Analyses using VL data should consider the findings of this study when planning analyses and controls related to disease processes or population incidence trajectories. Surveillance agencies should note that accurate surveillance by municipality is important because measuring incidence by state alone does not offer an equivalent characterization, and while there do exist small areas with incidences that can describes those of the others, nationwide surveillance at high resolution remains important to consider likely heterogeneity of processes contributing to VL burden. This applies to other diseases with incidences that depend on the scale and resolution of surveillance, which should be examined to assure whether this dependence does exist.

Acknowledgements: The authors thank the Brazilian Ministries of Health for allowing the use of the Visceral Leishmaniasis data for this study. The authors also acknowledge the resources of the Minnesota Supercomputing Institute for computational aid.

Author contributions:

Conceptualization: Joseph L Servadio, Gustavo Machado, Julio Alvarez, Matteo Convertino

Data Curation: Francisco Edilson de Ferreira Lima Júnior, Renato Vieira Alves

Formal Analysis: Joseph L Servadio

Funding Acquisition: Julio Alvarez, Matteo Convertino

Investigation: Francisco Edilson de Ferreira Lima Júnior, Renato Vieira Alves

Methodology: Joseph L Servadio

Project Administration: Matteo Convertino

Resources: Gustavo Machado, Julio Alvarez, Francisco Edilson de Ferreira Lima Júnior, Renato Vieira Alves

Software: Joseph L Servadio

Supervision: Matteo Convertino

Validation: Joseph L Servadio

Visualization: Joseph L Servadio

Writing – Original Draft Preparation: Joseph L Servadio, Gustavo Machado, Julio Alvarez, Matteo Convertino

Writing – Review & Editing: Joseph L Servadio, Gustavo Machado, Julio Alvarez, Francisco Edilson de Ferreira Lima Júnior, Renato Vieira Alves, Matteo Convertino

Competing interests: The authors do not have any competing interests.

References

1. Nsubuga P, White ME, Thacker SB, Anderson MA, Blount SB, Broome C V, et al. Public health surveillnace: A tool for targeting and monitoring interventions. In: Disease Control Priotiries in Developing Countries. 2nd ed. Washington, DC: The International Bank for Reconstruction and Development/The World Bank; 2006. p. 997–1015.
2. Hitchcock P, Chamberlain A, Van Wagoner M, Inglesby T V., O'Toole T. Challenges to Global Surveillance and Response to Infectious Disease Outbreaks of International Importance. Biosecurity Bioterrorism Biodefense Strateg Pract Sci [Internet]. 2007 Sep 28 [cited 2019 Jun 13];5(3):206–27. Available from: http://www.liebertpub.com/doi/10.1089/bsp.2007.0041
3. Pigott DM, Bhatt S, Golding N, Duda KA, Battle KE, Brady OJ, et al. Global distribution maps of the leishmaniases. Elife. 2014 Jun 27;3:e02851.
4. Linard C, Tatem AJ. Large-scale spatial population databases in infectious disease research. Int J Health Geogr [Internet]. 2012 [cited 2018 Jul 22];11:7. Available from: http://www.ij-healthgeographics.com/content/11/1/7
5. Levin SA. The Problem of Pattern and Scale in Ecology: The Robert H. MacArthur Award Lecture. Ecology [Internet]. 1992 Dec 1 [cited 2019 Apr 15];73(6):1943–67. Available from: http://doi.wiley.com/10.2307/1941447
6. Todd ECD. Challenges to global surveillance of disease patterns. Mar Pollut Bull [Internet]. 2006 Jan 1 [cited 2019 Jun 13];53(10–12):569–78. Available from: https://www.sciencedirect.com/science/article/pii/S0025326X06002931
7. Nsubuga P, Nwanyanwu O, Nkengasong JN, Mukanga D, Trostle M. Strengthening public health surveillance and response using the health systems strengthening agenda in developing countries. BMC Public Health [Internet]. 2010 Dec 3 [cited 2019 Jun 13];10(Suppl 1):S5. Available from: http://bmcpublichealth.biomedcentral.com/articles/10.1186/1471-2458-10-S1-S5
8. Piantadosi S, Byar DP, Green SB. The ecological fallacy. Am J Epidemiol. 1988;127(5):893–904.

9. Santos-Vega M, Martinez PP, Pascual M. Climate forcing and infectious disease transmission in urban landscapes: Integrating demographic and socioeconomic heterogeneity. Ann N Y Acad Sci. 2016;1382(1):44–55.

10. Mills HL, Riley S. The Spatial Resolution of Epidemic Peaks. PLoS Comput Biol. 2014 Apr 10;10(4):e1003561.

11. Pell B, Phan T, Rutter EM, Chowell G, Kuang Y. Simple multi-scale modeling of the transmission dynamics of the 1905 plague epidemic in Bombay. Math Biosci [Internet]. 2018 Jul [cited 2018 Jul 22];301:83–92. Available from: http://www.ncbi.nlm.nih.gov/pubmed/29673967

12. Jones SG, Kulldorff M. Influence of Spatial Resolution on Space-Time Disease Cluster Detection. PLoS One. 2012;7(10):e48036.

13. Mandelbrot B. How long is the coast of britain? Statistical self-similarity and fractional dimension. Science [Internet]. 1967 May 5 [cited 2019 Apr 16];156(3775):636–8. Available from: http://www.ncbi.nlm.nih.gov/pubmed/17837158

14. Peters EE. Fractal structures in the capital markets. Financ Anal J. 1989;45(4):32–7.

15. Camacho J, Guimerà R, Nunes Amaral LA. Robust Patterns in Food Web Structure. 2002 [cited 2019 Jan 5]; Available from: https://amaral.northwestern.edu/media/publication_pdfs/Camacho-2002-Phys.Rev.Lett.-88-228102.pdf

16. Kim H, Smith HB, Mathis C, Raymond J, Walker SI. Universal scaling across biochemical networks on Earth. Sci Adv. 2019 Jan 16;5(1):eaau0149.

17. Peng CK, Havlin S, Hausdorff JM, Mietus JE, Stanley HE, Goldberger AL. Fractal mechanisms and heart rate dynamics. Long-range correlations and their breakdown with disease. J Electrocardiol [Internet]. 1995 [cited 2019 Jan 5];28 Suppl:59–65. Available from: http://www.ncbi.nlm.nih.gov/pubmed/8656130

18. Schneeberger A, Mercer CH, Gregson SAJ, Ferguson NM, Nyamukapa CA, Anderson RM, et al. Scale-free networks and sexually transmitted diseases: a description of observed patterns of sexual contacts in Britain and Zimbabwe. Sex Transm Dis [Internet]. 2004 Jun [cited 2019 Jan

5];31(6):380–7. Available from: http://www.ncbi.nlm.nih.gov/pubmed/15167650
19. Small M, Walker DM, Tse CK. Scale-Free Distribution of Avian Influenza Outbreaks. Phys Rev Lett [Internet]. 2007 Oct 30 [cited 2019 Jan 5];99(18):188702. Available from: https://link.aps.org/doi/10.1103/PhysRevLett.99.188702
20. Convertino M, Liu Y, Hwang H. Optimal surveillance network design: a value of information model. Complex Adapt Syst Model [Internet]. 2014 Dec 27 [cited 2019 Apr 15];2(1):6. Available from: https://casmodeling.springeropen.com/articles/10.1186/s40294-014-0006-8
21. Ozgen VC, Kong W, Blanchard AE, Liu F, Lu T. Spatial interference scale as a determinant of microbial range expansion. Sci Adv. 2018 Nov 21;4(11):eaau0695.
22. Anversa L s., Tiburcio MGS, Richini-Pereira V nia B, Ramirez LE. Human leishmaniasis in Brazil: A general review. Vol. 64, Revista da Associacao Medica Brasileira. Associacao Medica Brasileira; 2018. p. 281–9.
23. World Health Organization. Leishmaniasis [Internet]. Fact Sheets. 2018 [cited 2018 Jul 22]. Available from: http://www.who.int/en/news-room/fact-sheets/detail/leishmaniasis
24. Bruhn FRP, Morais MHF, Cardoso DL, Bruhn NCP, Ferreira F, Rocha CMBM. Spatial and temporal relationships between human and canine visceral leishmaniases in Belo Horizonte, Minas Gerais, 2006–2013. Parasit Vectors [Internet]. 2018 Dec 28 [cited 2018 Jul 22];11(1):372. Available from: http://www.ncbi.nlm.nih.gov/pubmed/29954428
25. Killick-Kendrick R. The biology and control of Phlebotomine sand flies. Clin Dermatol [Internet]. 1999 May 1 [cited 2018 Jul 31];17(3):279–89. Available from: https://www.sciencedirect.com/science/article/pii/S0738081X99000462?via%3Dihub
26. Werneck GL. Visceral leishmaniasis in Brazil: rationale and concerns related to reservoir control. Rev Saude Publica [Internet]. 2014 Oct [cited 2018 Jul 22];48(5):851–6. Available from: http://www.ncbi.nlm.nih.gov/pubmed/25372177
27. Maia-Elkhoury ANS, Alves WA, Sousa-Gomes ML de, Sena JM de, Luna EA. Visceral leishmaniasis in Brazil: trends and challenges. Cad Saude Publica [Internet]. 2008 Dec [cited 2018

Jul 22];24(12):2941–7. Available from: http://www.ncbi.nlm.nih.gov/pubmed/19082286

28. Druzian AF, Souza AS de, Campos DN de, Croda J, Higa MG, Dorval MEC, et al. Risk Factors for Death from Visceral Leishmaniasis in an Urban Area of Brazil. Carvalho EM, editor. PLoS Negl Trop Dis [Internet]. 2015 Aug 14 [cited 2018 Jul 22];9(8):e0003982. Available from: http://www.ncbi.nlm.nih.gov/pubmed/26274916

29. World Health Organization. Leishmaniasis - Magnitude of the problem [Internet]. WHO. World Health Organization; 2014 [cited 2018 Jul 22]. Available from: http://www.who.int/leishmaniasis/burden/magnitude/burden_magnitude/en/

30. Ministério da Saúde Secretaria de Vigilância em Saúde Coordenação-Geral de Desenvolvimento da Epidemiologia emServiçosBrasil. Guia de Vigilância em Saúde : volume único [recurso eletrônico] . 3rd ed. 2019 [cited 2020 Apr 16]. 512–513 p. Available from: http://bvsms.saude.gov.br/bvs/publicacoes/guia_vigilancia_saude_3ed.pdf

31. Ministério da Saúde Secretaria de Vigilância em Saúde. Casos confirmados de Leishmaniose Visceral, Brasil, Grandes Regiões e Unidades Federadas. 1990 a 2018 [Internet]. 2019 [cited 2020 Apr 16]. Available from: https://www.saude.gov.br/images/pdf/2019/outubro/14/LV-Casos.pdf

32. Machado G, Alvarez J, Bakka HC, Perez A, Donato LE, de Ferreira Lima Júnior FE, et al. Revisiting area risk classification of visceral leishmaniasis in Brazil. BMC Infect Dis. 2019 Dec 3;19(1):2.

33. Instituto Brasileiro de Geografia Estastistica. Population Estimates [Internet]. 2018 [cited 2018 Aug 1]. Available from: https://www.ibge.gov.br/estatisticas-novoportal/sociais/populacao/9103-estimativas-de-populacao.html?edicao=17283&t=downloads

34. Alshkaki RSA. On the Zero-One Inflated Poisson Distribution. Int J Stat Distrib Appl. 2016 Dec 102(4):42–8.

35. Narula SC, Wellington JF. The Minimum Sum of Absolute Errors Regression: A State of the Art Survey. Int Stat Rev / Rev Int Stat. 1982 Dec;50(3):317.

36. Kirkpatrick S, Gelatt CD, Vecchi MP. Optimization by simulated annealing. Science (80-).

1983;220(4598):671–80.

37. Cover TM, Thomas JA. Elements of Information Theory. 2nd ed. Hoboken, NJ: John Wiley & Sons; 2006.

38. Convertino M, Muñoz-Carpena R, Kiker GA, Perz SG. Design of optimal ecosystem monitoring networks: hotspot detection and biodiversity patterns. Stoch Environ Res Risk Assess. 2015 May 14;29(4):1085–101.

39. R Core Team. R: A language and environment for statistical computing. Vienna, Austria: R foundation for statistical computing; 2019. Available from: https://www.r-project.org

40. Gillespie CS. Fitting heavy tailed distributions: The powerlaw package. J Stat Softw. 2015 Feb 1;64(2):1–16.

41. Sevá A da P, Mao L, Galvis-Ovallos F, Tucker Lima JM, Valle D. Risk analysis and prediction of visceral leishmaniasis dispersion in São Paulo State, Brazil. Carvalho EM, editor. PLoS Negl Trop Dis [Internet]. 2017 Feb 6 [cited 2019 Feb 11];11(2):e0005353. Available from: https://dx.plos.org/10.1371/journal.pntd.0005353

42. Campos R, Santos M, Tunon G, Cunha L, Magalhães L, Moraes J, et al. Epidemiological aspects and spatial distribution of human and canine visceral leishmaniasis in an endemic area in northeastern Brazil. Geospat Health. 2017 May 11;12(1):503.

43. Afonso MM dos S, Chaves SA de M, Magalhães M de AFM, Gracie R, Azevedo C, de Carvalho BM, et al. Ecoepidemiology of American Visceral Leishmaniasis in Tocantins State, Brazil: Factors Associated with the Occurrence and Spreading of the Vector Lutzomyia (Lutzomyia) longipalpis (Lutz & Neiva, 1912) (Diptera: Psychodidae: Phlebotominae). In: The Epidemiology and Ecology of Leishmaniasis [Internet]. InTech; 2017 [cited 2019 Feb 11]. Available from: http://www.intechopen.com/books/the-epidemiology-and-ecology-of-leishmaniasis/ecoepidemiology-of-american-visceral-leishmaniasis-in-tocantins-state-brazil-factors-associated-with

44. Reis LL dos, Balieiro AA da S, Fonseca FR, Gonçalves MJF, Reis LL dos, Balieiro AA da S, et al.

Leishmaniose visceral e sua relação com fatores climáticos e ambientais no Estado do Tocantins, Brasil, 2007 a 2014. Cad Saude Publica [Internet]. 2019 Jan 10 [cited 2019 Feb 11];35(1). Available from: http://www.scielo.br/scielo.php?script=sci_arttext&pid=S0102-311X2019000105004&lng=pt&tlng=pt

45. Tejedor A, Singh A, Zaliapin I, Densmore AL, Foufoula-Georgiou E. Scale-dependent erosional patterns in steady-state and transient-state landscapes. Sci Adv. 2017 Sep 1;3(9):e1701683.

46. Lima ÁLM, de Lima ID, Coutinho JF V, de Sousa ÚPST, Rodrigues MAG, Wilson ME, et al. Changing epidemiology of visceral leishmaniasis in northeastern Brazil: a 25-year follow-up of an urban outbreak. Trans R Soc Trop Med Hyg. 2017;111(10):440–7.

47. Roy M, Zinck RD, Bouma MJ, Pascual M. Epidemic cholera spreads like wildfire. Sci Rep. 2014 Jan 15;4:3710.

48. Kinsley AC, Patterson G, VanderWaal KL, Craft ME, Perez AM. Parameter values for epidemiological models of foot-and-mouth disease in Swine. Front Vet Sci. 2016 Jun 1;3(JUN).

49. de Carvalho IPSF, Peixoto HM, Romero GAS, de Oliveira MRF. Cost of visceral leishmaniasis care in Brazil. Trop Med Int Heal. 2017 Dec 1;22(12):1579–89.

50. Reis LL dos, Balieiro AA da S, Fonseca FR, Gonçalves MJF. Changes in the epidemiology of visceral leishmaniasis in Brazil from 2001 to 2014. Rev Soc Bras Med Trop. 2017 Sep;50(5):638–45.

51. World Health Organization. Number of cases of visceral leishmaniasis reported - Data by country [Internet]. WHO. World Health Organization; 2018 [cited 2019 Feb 11]. Available from: http://apps.who.int/gho/data/node.main.NTDLEISHVNUM?lang=en

52. World Health Organization. Brazil [Internet]. [cited 2019 Feb 11]. Available from: https://www.who.int/leishmaniasis/resources/BRAZIL.pdf

53. Wheeler JG, Sethi D, Cowden JM, Wall PG, Rodrigues LC, Tompkins DS, et al. Study of infectious intestinal disease in England: Rates in the community, presenting to general practice, and reported to national surveillance. Br Med J. 1999 Apr 17;318(7190):1046–50.

54. Undurraga EA, Halasa YA, Shepard DS. Use of Expansion Factors to Estimate the Burden of Dengue in Southeast Asia: A Systematic Analysis. PLoS Negl Trop Dis. 2013;7(2).

55. Singh SP, Reddy DCS, Rai M, Sundar S. Serious underreporting of visceral leishmaniasis through passive case reporting in Bihar, India. Trop Med Int Heal. 2006 Jun;11(6):899–905.

56. Sadeq M. Spatial patterns and secular trends in human leishmaniasis incidence in Morocco between 2003 and 2013. Infect Dis Poverty. 2016;5(1).

57. Dewan A, Abdullah AYM, Shogib MRI, Karim R, Rahman MM. Exploring spatial and temporal patterns of visceral leishmaniasis in endemic areas of Bangladesh. Trop Med Health. 2017 Nov 15;45(1).

Supplemental material captions

S1 Table. Frequency of incidence values by municipality within each state and by state for the nation.

S2 Table. Best fitting parameterizations for each considered distribution and associated sum of absolute error (SAE) values.

S3 File. Original data with annual Visceral Leishmaniasis incidence by municipality in Brazil, 2004 – 2014 with R code used for analysis.

Fig 1

Click here to access/download;Figure;Fig1_Rev.tiff

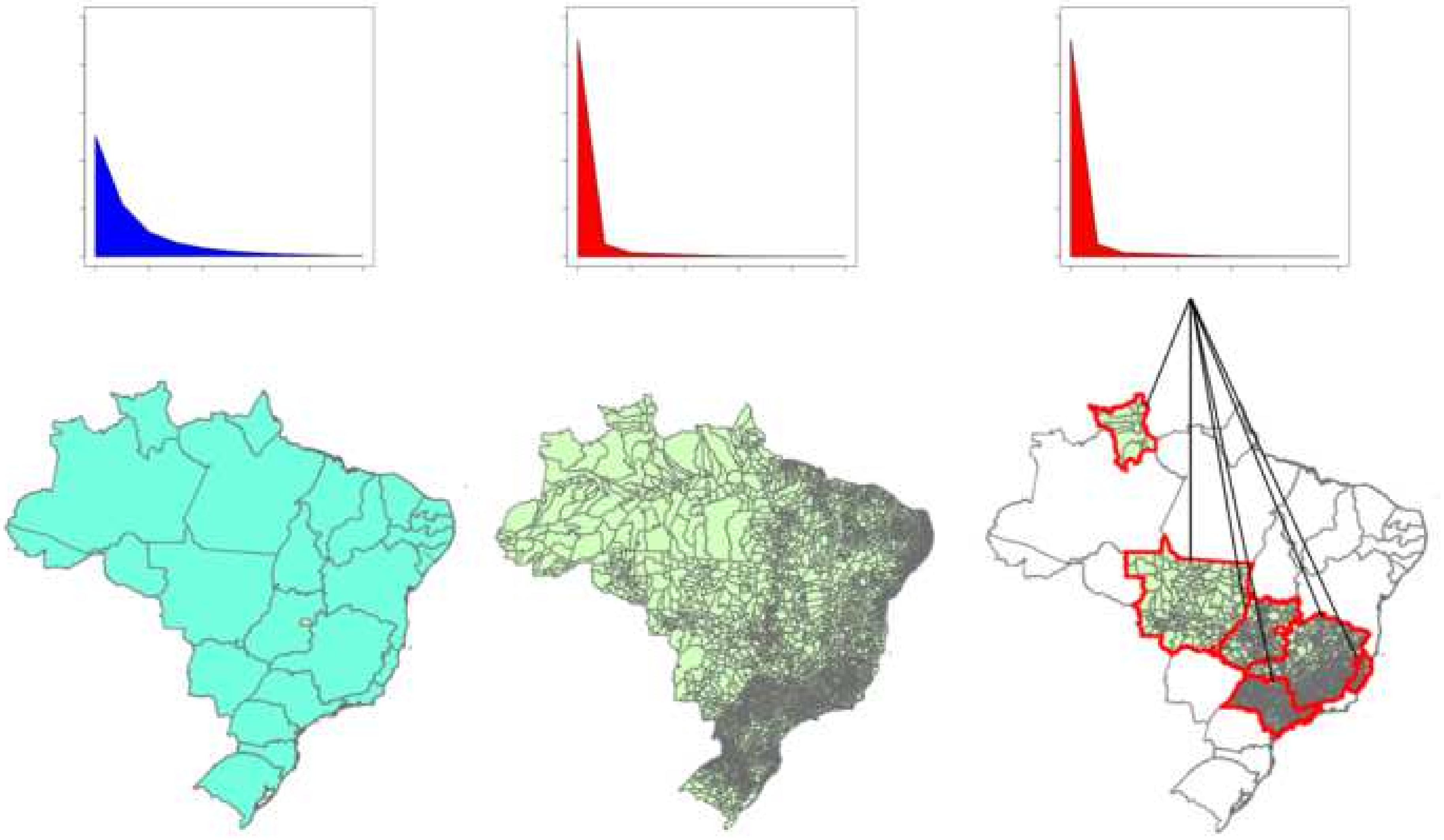

(a) (b) (c)

Fig 2

Click here to access/download;Figure;Fig2.tiff

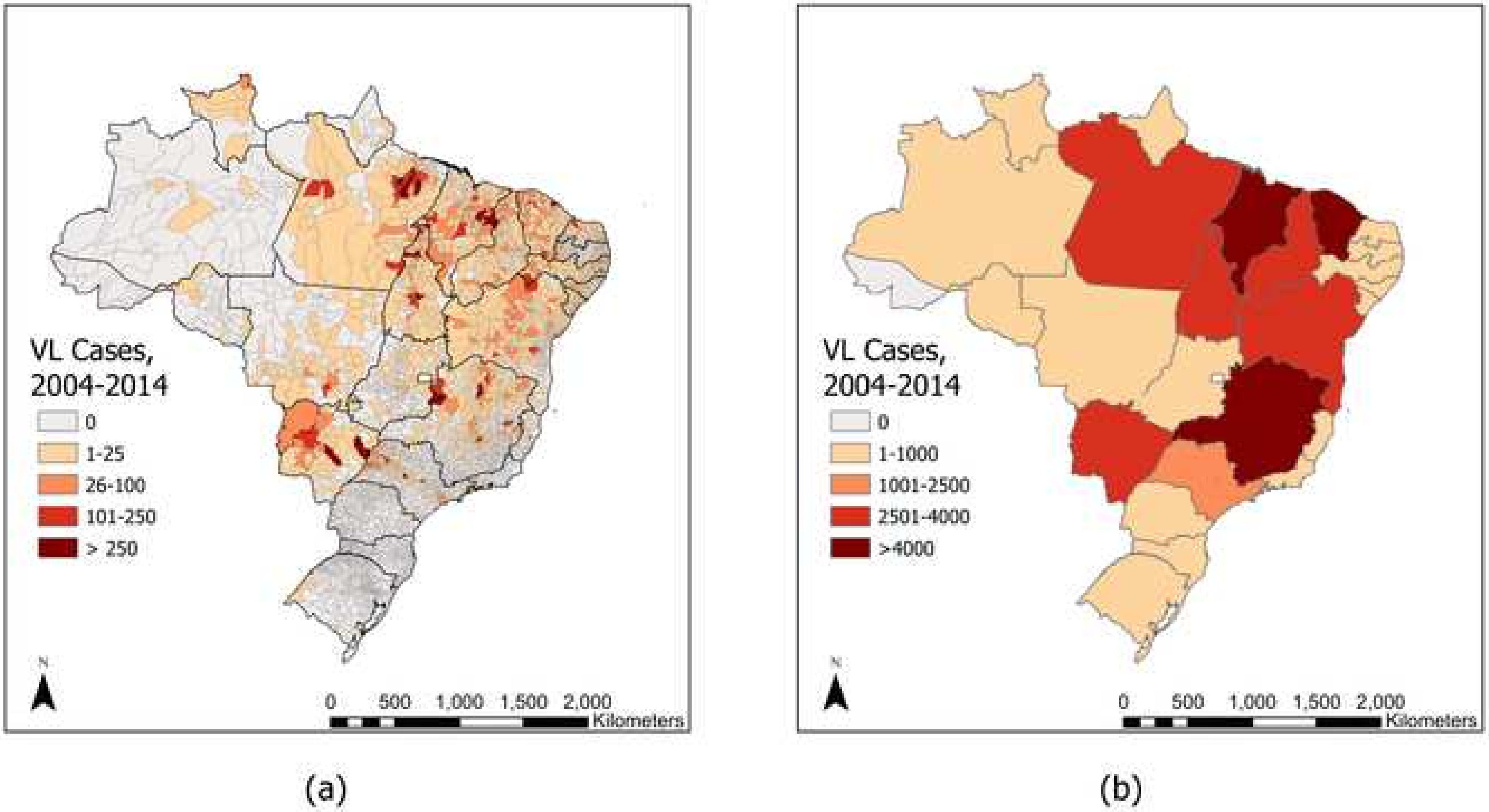

Fig 3

Click here to access/download;Figure;Fig3.tiff

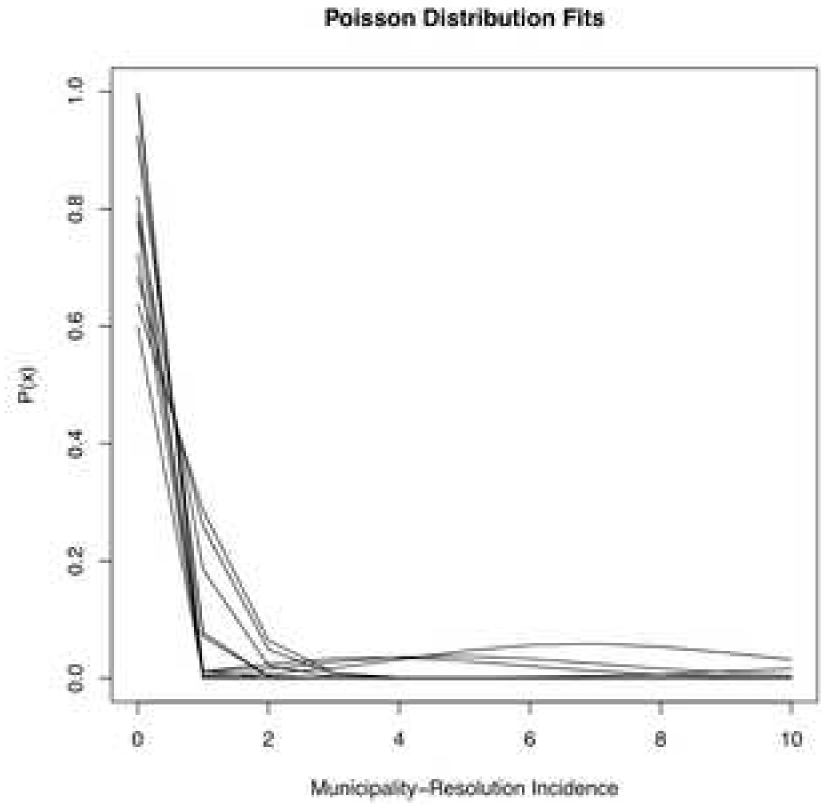

(a)

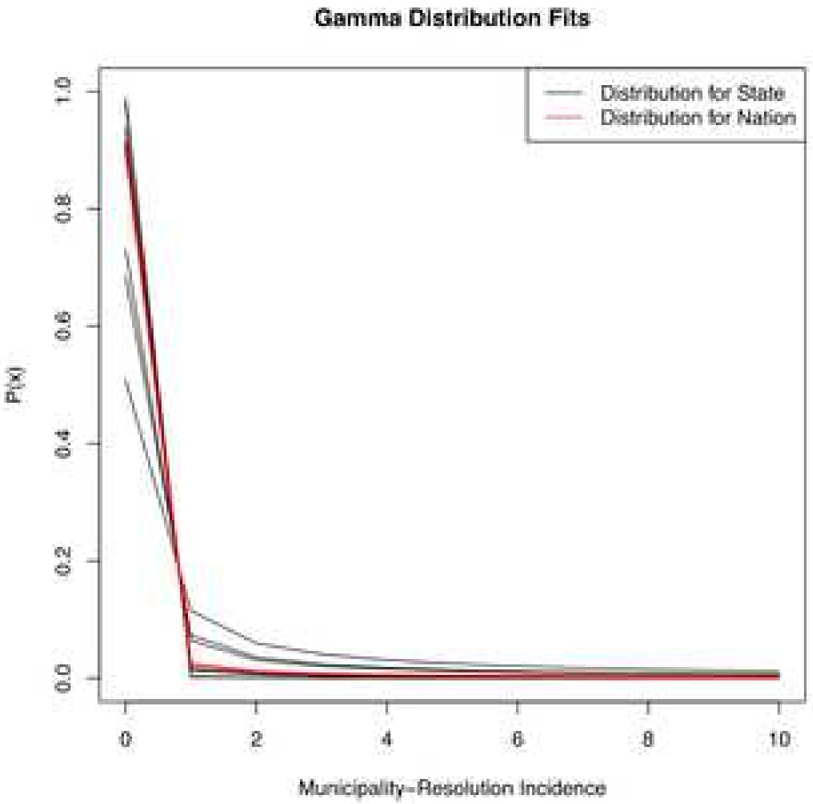

(b)

 Click here to access/download;Figure;Fig4.tiff

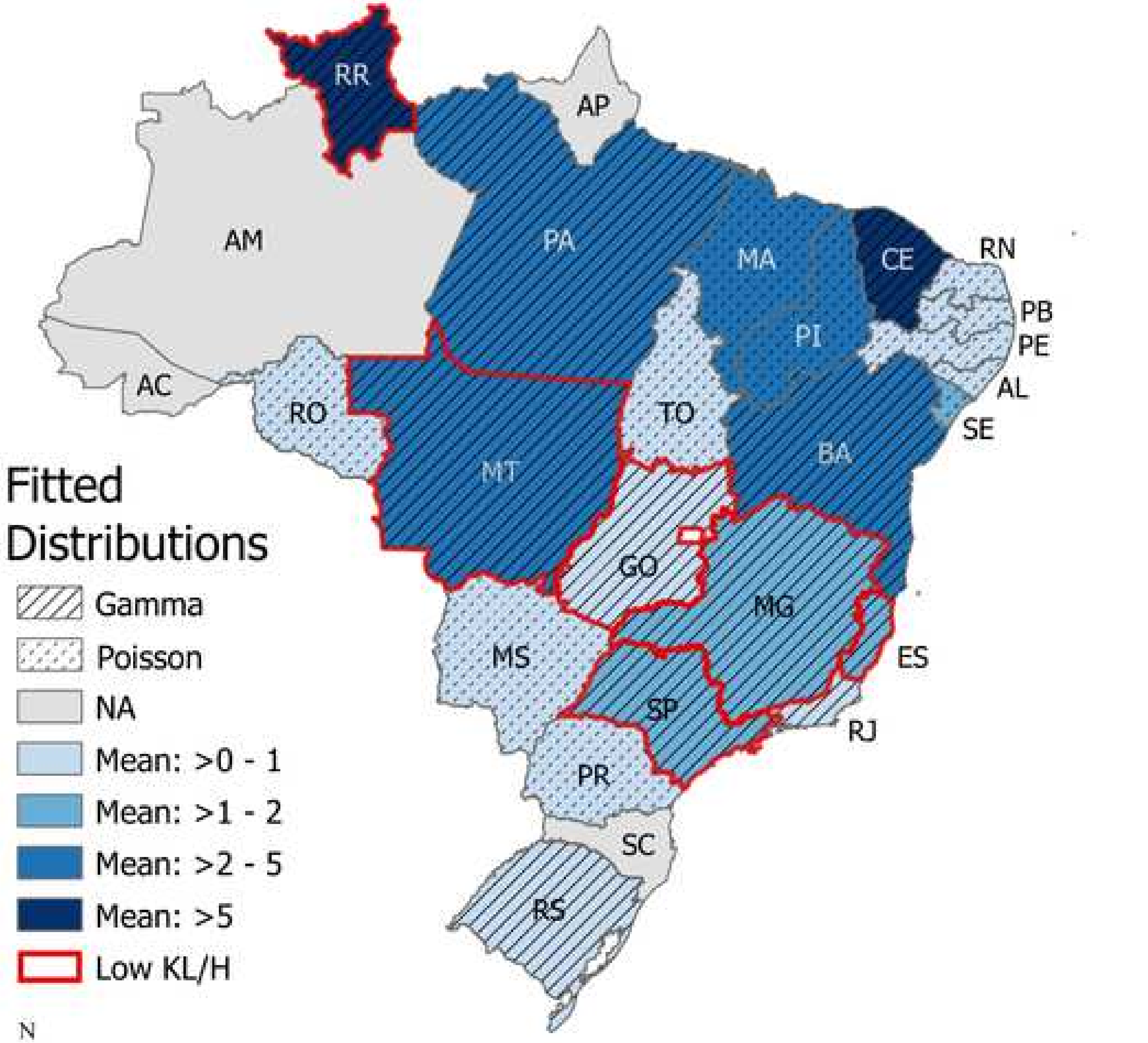

S1 Table

Click here to access/download
Supporting Information
S1_Table.csv

S2 Table

Click here to access/download
Supporting Information
S2_Table.xlsx

Click here to access/download
Supporting Information - Compressed/ZIP File Archive
S3_File.zip

Information differences across spatial resolutions and scales for disease surveillance and analysis: The case of Visceral Leishmaniasis in Brazil

Short title: Sensitivity to spatial resolution and scale for infectious disease surveillance

Joseph L Servadio,[1]* Gustavo Machado,[2] Julio Alvarez,[3, 4] Francisco Edilson de Ferreira Lima Júnior,[5] Renato Vieira Alves,[5] Matteo Convertino[6]

[1] Division of Environmental Health Sciences, University of Minnesota School of Public Health, 420 Delaware Street SE, Minneapolis, MN, USA.

[2] Department of Population Health and Pathobiology, College of Veterinary Medicine, North Carolina State University, 1060 William Moore Drive, Raleigh, NC, USA.

[3] VISAVET Health Surveillance Center, Universidad Complutense, Avda Puerta de Hierro S/N, 28040 Madrid, Spain.

[4] Departamento de Sanidad Animal, Facultad de Veterinaria, Universidad Complutense, Avda Puerta de Hierro S/N, 28040 Madrid, Spain

[5] Secretaria de Vigilância em Saúde, Ministério da Saúde (SVS-MH), Brasília, Brazil

[6] Nexus Group, Graduate School of Information Science and Technology and GI-CoRE Station for Big-Data and Cybersecurity, Hokkaido University, Sapporo, Hokkaido, Japan.

* Corresponding author: Joseph L Servadio. Division of Environmental Health Sciences, University of Minnesota School of Public Health, MMC807, 420 Delaware Street SE, Minneapolis, MN, 55455, USA. serva024@umn.edu

Abstract

Nationwide disease surveillance at a high spatial resolution is desired for many infectious diseases, including Visceral Leishmaniasis. Statistical and mathematical models using data collected from surveillance activities often use a spatial resolution and scale either constrained by data availability or chosen arbitrarily. Sensitivity of model results to the choice of spatial resolution and scale is not, however, frequently evaluated. This study aims to determine if the choice of spatial resolution and scale are likely to impact statistical and mathematical analyses. Visceral Leishmaniasis in Brazil is used as a case study. Probabilistic characteristics of disease incidence, representing a likely outcome in a model, are compared across spatial resolutions and scales. Best fitting distributions were fit to annual incidence from 2004 to 2014 by municipality and by state. Best fits were defined as the distribution family and parameterization minimizing the sum of absolute error, evaluated through a simulated annealing algorithm. Gamma and Poisson distributions provided best fits for incidence, both among individual states and nationwide. Comparisons of distributions using Kullback-Leibler divergence shows that incidence by state and by municipality do not follow distributions that provide equivalent information. Few states with Gamma distributed incidence follow a distribution closely resembling that for national incidence. These results demonstrate empirically how choice of spatial resolution and scale can impact mathematical and statistical models.

Keywords: Visceral Leishmaniasis; surveillance; scale; resolution; distributions; KL divergence

1. Introduction

Infectious disease research often relies on data generated through passive or active surveillance activities, which can suffer from important limitations due to variation in methods and capacities for data collection [1, 2]. Typically, researchers aim to collect data at a high spatial resolution, that is, in the form of small surveillance units such as counties or municipalities rather than states or nations [3, 4], though this may not always be seen as beneficial [5]. Conducting surveillance at a high spatial resolution, however, is often unrealistic when considering large areas and constrained resources [6, 7].

Data collected from infectious disease surveillance activities are often used in researching involving mathematical or statistical models. In such analyses, matters related to data quality are of concern. The choice of spatial resolution is often based on data availability or chosen arbitrarily, with little attention given to whether this decision may impact model results. Aggregating data into larger spatial units can aid in computational efficiency, but creates the risk of introducing ecological fallacy [8] and masking heterogeneity within those larger units if conclusions are drawn inappropriately [9–11]. This would be particularly problematic when aiming to seek disease etiologies. Previous studies using mathematical or statistical models have investigated the importance of high resolution data by repeating analyses using data at different resolutions and then comparing results [10–12].

An additional challenge to high quality surveillance is the need for surveillance over a large spatial scale, referring to the entire area where surveillance is being conducted. Here, spatial scale differs from spatial resolution in their definitions as follows: spatial scale refers to the total spatial area being examined, while spatial resolution refers to the size of the individual spatial units within that area. Large-scale surveillance can be particularly challenging for nations with large land areas and populations. In these circumstances, there is potential benefit in identifying a smaller area, such as a state or group of states, where surveillance can adequately estimate the national disease burden. The characteristic of having smaller

areas representative of the whole for a large range of sizes is known as scale invariance or fractality [13]. Scale invariance is ubiquitous in many socio-ecological patterns such as finance [14], ecology [15], biochemical processes [16], and biology across time scales [17].

Scale invariance is an infrequently examined concept in infectious disease surveillance and epidemiology in general, though it has relevance to many forms of data analysis or modeling. Scale invariance in infectious disease research is more frequently used to describe scale-free networks, typically applied to human communicable diseases [18] or transmission paths of infectious diseases [19]. For practical purposes in epidemiology, identifying smaller regions that represent a larger area or even an entire nation could allow the design of targeted surveillance strategies and conserve resources [20]. Even in the absence of true scale invariance, self-similarity can be observed [13] where some smaller areas can be used to describe the whole. In other situations, however, the spatial scale of interest impacted the physical processes being studied [21].

The topics of spatial resolution and scale are relevant for research pertaining to numerous health outcomes. Here, Visceral Leishmaniasis is examined as a case study. Visceral Leishmaniasis (VL), caused by a *Leishmania infantum* parasite (known in Latin America as *Leishmania chagasi*) [22], is the most severe form of Leishmaniasis [23] and is fatal in the vast majority of untreated cases [24]. The parasite is typically transmitted from an infected to a non-infected host through the bites of phlebotomine sand flies [25]. Visceral Leishmaniasis presentation can include symptoms such as fever, enlargement of the spleen and liver, and anaemia [23]. Worldwide, there are between 50,000 and 90,000 estimated VL cases yearly [23].

Brazil is one of ten world nations with the greatest VL burdens [23], with the remaining nations located primarily in East Africa and South and Southeast Asia [23]. Estimates report that 90% of VL cases that occur in the Americas occur in Brazil [23] where~~, particularly in urban regions~~, canines are a disease

reservoir [24, 26, 27]. Areas of Brazil that previously had accounted for only 15% of all cases reported nationally now can see nearly half of the nation's cases [27]. The disease has also become more common in urban areas in recent years [25, 27, 28]decades, [26, 28, 29], making it a major public health concern and an important target for surveillance programs. As of 2015, based on the data used for this study, the Federal District and 18 of the 26 states in Brazil meet the criterion of being an endemic state for VL, which is seeing at least one case in all three previous years [30]. The states that were not endemic at the time are Acre, Amapá, Amazonas, Espírito Santo, Paraná, Rondônia, Roraima, and Santa Catarina [30, 31]. As of 2019, Espírito Santo and Paraná became endemic states [31].

Field Code Changed

This study aims to assess the potential impact of using different spatial resolutions and scales on statistical and mathematical models using surveillance data applied to VL cases in Brazil. In order to do so, two objectives are pursued: (1) to determine if surveillance using incidence by state or municipality leads to different conclusions regarding disease distribution; and (2) to determine if conducting VL surveillance on a region within Brazil would adequately characterize the nation's VL incidence. This is done by using best fitting probability distributions to describe disease data without incorporating outside information. A conceptual visualization of the study aims are presented in Figure 1. Prior to conducting statistical analyses or models, researchers may need to decide whether to consider data using different spatial resolutions as well as the scale of analysis; the results of this study will provide insight into whether the subsequent results may be sensitive to this decision.

Fig 1. Graphical overview of the study objectives: (a) fit distribution to annual incidence of Visceral Leishmaniasis (VL) by state, (b) fit distribution to annual incidence of VL by municipality, (c) fit distributions to annual incidence of VL by municipality within each state. Comparisons of these fitted distributions indicate whether characterizing VL incidence by state or municipality are equivalent, impacting statistical analyses using these data.

2. Methods and Materials

2.1. Study setting and data

The setting for this study is Brazil, the largest nation in South America in both land area and population. Case data were provided by the Brazilian Ministries of Health [32] and include VL case counts by municipality nationwide, with the exception of the Federal District, totaling 26 states and 5,561 municipalities. The Federal District was excluded because it is not a state with multiple municipalities, and therefore cannot be aggregated to differentiate between the municipality resolution and the state resolution. Yearly case counts by municipality between 2004 and 2014 are reported. Annual populations for each municipality are publicly available through the Brazilian Institute of Geography and Statistics [33] to calculate annual incidence, discretized to represent cases per 100,000 population per year. Population data were available for all years except 2007 and 2010. In these two years, the arithmetic means of the populations of the two adjacent years were used in place of the missing populations. ~~A total of 5,538 municipalities were matched to population data~~Population data were available for 5,538 of the municipalities with VL data, providing the final sample for this study.

2.2~~1~~ Inferring probability distributions

This study compares spatial resolutions and scales using probability distributions rather than by fitting models with assumptions and conducting a sensitivity analysis. Best fitting distributions from multiple considered distributional families were fit for (1) annual incidence for each individual state using the municipality as the unit of surveillance; (2) annual incidence nationwide using the municipality as the unit of surveillance; and (3) annual incidence nationwide using the state as the unit of surveillance (Figure 1). All 11 years of observation were included.

Common candidate distributions were selected based on exploratory analyses and suitability for discrete incidence data, and wide ranges of parameters for each distribution were tested. The Poisson, Zero Inflated Poisson (ZIP), and Zero One Inflated Poisson (ZOIP) [34] distributions were selected as candidate distributions along with the Gamma, Exponential, Power Law, and Uniform distributions rounded to fit discrete data. These are described in Table 1.

Table 1. Candidate distributions used for fitting distributions

Distribution	Probability mass function
Poisson	$P(X = x) = \frac{\theta^x}{x!} \exp(-\theta)$
Zero Inflated Poisson (ZIP)	$P(X = 0) = \alpha + (1-\alpha) \exp(-\theta)$<br>$P(X = x) = (1-\alpha) \frac{\theta^x}{x!} \exp(-\theta), x > 1$
Zero One Inflated Poisson (ZOIP)	$P(X = 0) = \alpha + (1-\alpha-\beta) \exp(-\theta)$<br>$P(X = 1) = \beta + (1-\alpha-\beta)\, \theta \exp(-\theta)$<br>$P(X = x) = (1-\alpha-\beta) \frac{\theta^x}{x!} \exp(-\theta), x > 1$
Rounded Exponential	$P(X = x) = \int_0^{x+0.5} f(y)\, dy - \int_0^{x-0.5} f(y)\, dy$<br>$f(y) = \theta \exp(-\theta\, y)$
Rounded Gamma	$P(X = x) = \int_0^{x+0.5} f(y)\, dy - \int_0^{x-0.5} f(y)\, dy$<br>$f(y) = \frac{\beta^\alpha}{\Gamma(\alpha)} y^{\alpha-1} \exp(-\beta\, y)$
Rounded Power Law	$P(X = x) = \int_0^{x+0.5} f(y)\, dy - \int_0^{x-0.5} f(y)\, dy$<br>$f(y) = \frac{\alpha - 1}{x_{min}} \left(\frac{x}{x_{min}}\right)^{-\alpha}, x > x_{min}$
Rounded Uniform	$P(X = x) = \frac{1}{b-a}, x \in \mathbb{Z} \cap [a,b]$

Each distribution was evaluated for the optimal parameter set that minimizes the sum of absolute error (SAE), defined as

$SAE = \sum_x |p(x) - p_{obs}(x)|$ (Equation 1)

where $p(x)$ represents the probability of observing an incidence of x cases per 100,000 person-years based on the proposed distribution indicated in Table 1 and $p_{obs}(x)$ represents the observed proportion of incidence values equaling x. This measure compares similarities between the proposed distributions and observed data and is less sensitive to outliers than other measures [35].

The optimal parameter set for each distribution was found through simulated annealing [36], an optimization algorithm based on Monte Carlo sampling. The algorithm was run with three chains for 50,000 simulations to assure convergence. Convergence was reached if each of the three chains produced identical parameter values, indicating a lack of movement to other parameter values, for at least 500 iterations, as well as one of the following hierarchical criteria: (1) final parameter values across pairs of chains had an absolute difference of less than 0.01; (2) final parameter values had an absolute difference of less than 0.1 and associated SAE values had an absolute difference of less than 0.01; (3) SAE values had an absolute difference of less than 0.001. The second and third criteria were necessary due to some parameterizations having very similar SAE values. If convergence was not reached in 50,000 iterations, the three chains were restarted with the parameterization that led to the lowest SAE value in the chain as initial values, and the simulated annealing algorithm was repeated, increasing the simulation count by 5,000. This was repeated until convergence was reached.

2.32 Comparing Distributions

2.32.1 Sensitivity to spatial resolution

The first aim of comparing distributions is to determine if different inferential conclusions may be reached using the municipality or the state as the resolution of nationwide surveillance. This was done by comparing the fitted state-resolution distribution to an expected state-resolution distribution for the nation based on the fitted municipality-resolution distribution for the nation. This expected distribution was

generated empirically by drawing Monte Carlo samples from the fitted municipality-resolution distribution.

If incidence is denoted by X as a random variable following the fitted municipality-resolution distribution for the nation; states are denoted by s; state s has n_s municipalities, denoted by m; and municipality m has a population of p_{my} in year y, this empirical distribution was generated by drawing 1,000 samples of

$$\frac{\sum_{m=1}^{s} p_{my} x_m}{\sum_{m=1}^{s} p_{my}} \text{ (Equation 2)}$$

for each state under each year of observation. The numerator of Equation 2 draws values of X_m for each municipality in a state and multiplies the value by the population of the municipality to sample a case count for the municipality. The sum of these is divided by the total state population to produce an expected state-resolution incidence for a year based on the municipality-resolution distribution for incidence.

Relative proportions of incidence values in these simulated values were compared to the probabilities of each incidence value from the fitted state-resolution distribution through Kullback-Leibler (KL) divergence [37]. KL divergence represents the additional information needed when using one distribution to describe data from another distribution. By measuring dissimilarity, it has an inverse relationship to Mutual Information, which represents similarity of variables [37]. Thus, KL divergence is a measure of the Value of Information [38]. For two random variables, denoted A and B, the KL divergence from A to B, compared to the Shannon entropy in the distribution of A, shows a relative increase in information, using bits as units, needed to describe the distribution of B with that of A [37]. This is shown in the ratio of KL divergence to Shannon entropy (denoted *H)*, which can be defined as the Required Relative Information Gain (RRIG), where

$$RRIG = \frac{KL_{A \to B}}{H_A} \text{ (Equation 3)}$$

shows the needed increase in information for the distribution of B to describe data from the distribution of A [37]. A value of 1 represents an information increase by 100%, or a doubling of information, though this is not an upper bound. A large RRIG value is indicative of distinct differences between distributions, indicating that characterizations of VL incidence are sensitive to the resolution of surveillance. An RRIG above 5% was selected as a threshold for having a distinct difference in distribution.

2.32.2 Sensitivity to spatial scale

The aim of comparing spatial scales involves comparing the municipality-resolution distributions of each state and of the nation. In the presence of scale invariance, individual states would have the same or similar distributions, which would be similar to the distribution for the nation. Distributions for municipality-resolution case counts for the nation and each state were compared through RRIG.

All analyses were performed using R version 3.6.0 [39]. The ‘poweRlaw’ package was used to calculate probability density and mass for the Power Law distribution [40].

3. Results

Of the 26 states in Brazil, 22 were included in analyses since they all observed more than five nonzero unique annual municipality-resolution incidence values over the study period (Table S1). The remaining four states were excluded because their incidences over the 11 years did not provide enough unique values to reliably fit a distribution. All 18 endemic states from 2015 [31] were included as well as Espírito

Santo, Paraná, Rondônia, and Roraima. Figure 2 (a and b) shows total case counts by state and by municipality over the entire time period.

Fig 2. Total case counts by (a) municipality and (b) state between 2004 and 2014

3.1 Fitted distributions

The uniform distribution for nationwide municipality-resolution incidence was not able to converge after increasing the iteration count to 200,000. All other distributions converged to optimal values. The best fitting municipality-resolution distributions for individual states varied. Annual incidence values from ten states were best fit by the rounded Gamma distribution, incidences from seven states were best fit by the Poisson distribution, incidences from three states were best fit by the Zero Inflated Poisson distribution, and incidences from two states were best fit by the Zero One Inflated Poisson distribution. Specific parameters are shown by state in Table 2. Plots of the probability mass functions of each state's municipality-resolution distribution are shown in Figure 3. Nationwide, the best fitting distribution for municipality-resolution incidence was the Gamma distribution, and the best fitting distribution for state-resolution incidence was the Zero One Inflated Poisson distribution (Table 2). No notable differences were seen in distributional fit among VL endemic and non-endemic states.

Table 2. Distributions for municipality-resolution incidences by state, municipality-resolution incidence nationwide, and state-resolution incidence nationwide. KL/H calculated from Equation 3 shows comparisons of fitted distributions to that for the nationwide municipality-resolution distribution (reference).

State	Distribution	Mean	Variance	KL/H
AL*	Poisson(0.234)	0.234	0.234	0.456

BA*	Gamma(0.078, 0.022)	3.545	161.157	0.191
CE*	Gamma(0.191, 0.038)	5.026	132.272	0.824
ES	Gamma(0.003, 0.002)	1.500	750.000	0.084
GO*	Gamma(0.016, 0.016)	1	62.5	0.009
MA*	ZOIP(0.599, 0.004, 7.292)	2.899	15.605	1.154
MG*	Gamma(0.024, 0.020)	1.2	60	0.001
MS*	Poisson(0.378)	0.378	0.378	0.791
MT*	Gamma(0.015, 0.003)	5	1666.667	0.007
PA*	Gamma(0.094, 0.022)	4.273	194.215	0.289
PB*	Poisson(0.077)	0.077	0.077	0.155
PE*	ZIP(0.819, 4.193)	0.759	3.365	0.263
PI*	ZIP(0.723, 14.44)	4.000	45.759	0.878
PR	Poisson(0.003)	0.003	0.003	0.151
RJ*	Gamma(0.002, 0.009)	0.222	24.691	0.108
RN*	Poisson(0.085)	0.085	0.085	0.165
RO	Poisson(0.013)	0.013	0.013	0.128
RR	Gamma(0.015, 0)	15	15000	0.021
RS*	ZIP(0.998, 4.338)	0.009	0.046	0.146
SE*	ZOIP(0.776, 0.006, 5.254)	1.151	5.43	0.388
SP*	Gamma(0.014, 0.013)	1.077	82.840	0.01
TO*	Poisson(0.450)	0.450	0.450	0.960
Brazil (Municipality)	Gamma(0.024, 0.017)	1.411	83.045	Reference
Brazil (State)	ZOIP(0.379, 0.074, 2.82)	1.617	3.353	0.425

* denotes states considered endemic for Visceral Leishmaniasis in 2015 [30, 31]

Fig 3. Probability mass functions for distributions fit to municipality-resolution incidences among states and for the nation. Incidences were fit by either (a) a Poisson distribution, including distributions with zero and one inflations, or (b) a Gamma distribution.

3.2 Comparisons across resolutions and scales

The RRIG from Equation 3 was used to quantify the similarities between distributions. The distributions fitted to state-resolution incidence and to the empirical distribution created from the national municipality-resolution distribution and Equation 2 were first compared to determine the sensitivity to the resolution of surveillance data. The RRIG was 0.425, representing a needed increase of information by 42.5% (Table 2). These results are indicative of strong sensitivity to the resolution of surveillance; the distribution for VL incidence by state does not accurately describe the distribution of incidence by municipality.

Comparisons between individual states' municipality-resolution incidence and national municipality-resolution incidence are shown in Table 2 using RRIG from Equation 3. The nationwide, municipality-resolution distribution is used as the reference for comparisons. The results show that six of the 22 states had incidence following a distribution close to that of the nation (RRIG<0.05) (Table 2, Figure 4). Any of these states could individually describe municipality-resolution incidence of the nation. Because not all states adequately characterize national burden, true scale invariance was not seen, though self-similarity was seen in the selected states. The states that exhibited some self-similar behaviors all followed a Gamma distribution with generally similar parameters, particularly low values of shape parameters. Many of these states were located near the center of the nation (Figure 4).

Fig 4. Expected values and families of fitted municipality-resolution distributions. States outlined in red had low KL divergence to Shannon entropy ratio with the national municipality-resolution distribution, indicating self-similarity.

4. Discussion

This study aimed to assess the importance of the spatial scale and resolution used for VL surveillance and subsequent quantitative analyses. This is also reflective of the dynamics of VL at different scales determined by the distributions of incidence. Probability distributions were fit to incidences at different spatial resolutions and scales and then compared to determine if distributional fit was sensitive to the choice of scale and resolution. Aggregating municipality-resolution incidences into state-resolution incidences led to notably different probabilistic characteristics of disease burden, suggesting the existence of different processes driving disease occurrence at the two resolutions. When continuing surveillance at the municipality resolution, six states' incidences follow distributions that adequately describe those of each other as well as the nation of Brazil. While our results provide evidence against true invariance to resolution and scale, some self-similarity is seen in both distributional parameters and moments. This happens for states that are following a Gamma distribution, which implies medium-long range dispersal of cases and a potential tendency toward a power-law distribution for small scale and shape parameters.

The self-similarity seen in six states does not indicate that significant resources can be saved in Brazil by concentrating surveillance in a smaller area because they are not representative of the other states. The remaining states still need to undergo surveillance in order for their VL burden to be adequately characterized. Furthermore, it is of interest for public health to know where all VL cases occur in order to intervene in an outbreak. If greater self-similarity were seen, it would largely be of interest to researchers who could potentially generalize results of a smaller area to the nation of Brazil through conducting more intensive data collection for additional data in a smaller area. However, because scale invariance was not

seen and self-similarity was seen in a small number of states, it is unlikely that descriptions of VL burden in a smaller region of Brazil are generalizable to the entire nation. These considerations consider the current observed state, for instance in case of a widespread propagation of the disease in long range.

Differences in municipality-resolution distributions among states suggest that different factors may influence VL risk across states. Environmental factors shown to influence VL case risk include vector populations, canine cases, precipitation [41], proximity to wooded areas [42], land use, deforestation [43], temperature, and humidity [44]. The question of resolution dependence is targeting whether the elementary unit at which we look into VL dynamics makes a difference for reproducing the distributional representation of VL incidence at the scale of analysis. These natural phenomena related to VL burden may differ across spatial scales and resolutions, similarly to other physical phenomena [45]. Though vector populations and precipitation do not explicitly conform to local political boundaries, different regions of Brazil likely see differences in these risk factors through differences in managing socio-ecological factors. These facts and the finding that distributions across states differ (also varying when resolution varies) are important considerations when analyzing disease data. It would be advisable to analyze data for individual locations [41, 44, 46] or use random effects [32].

The results from this study do not necessarily suggest that one spatial resolution is more “correct” than another or favor a particular resolution for analysis. The resolution for statistical analyses should rely on the research question being posed and desired interpretation of results. However, the resolution dependence implies that, assuming accuracy and precision in assigning municipalities to observed cases, aggregating incidence to the state resolution likely introduces ecological fallacy. Thus, high resolution is likely beneficial to capture disease dynamics accurately. For high-resolution incidence, the most likely VL dynamics are represented by the Gamma distribution. These considerations should be always taken into account when collecting and analyzing data because they indicate that the choice of resolution will impact model results and their interpretation. Data characterizations and analyses at one resolution are not

interchangeable for characterizations and analyses at the other resolution. A related point to note is that diligent surveillance is important when conducted at a finer spatial resolution to ensure accuracy of municipalities that are matched to cases.

This study is, to the authors' knowledge, the first to examine VL incidence for sensitivity to scale and resolution of surveillance data by finding best-fitting distributions to characterize incidence. Other studies have analyzed the fractality of other diseases, such as cholera, and how that is important for a simple estimation of disease spread in term of geography and magnitude [47]. Similar distribution fitting processes are used in veterinary epidemiology [48], but less frequently in human disease epidemiology. This analysis is important for informing future disease burden by providing location-specific estimates of expected annual incidence.

The findings of this study can benefit surveillance, healthcare infrastructure, digital epidemiology, and public health research focused on disease ecology. Care for an individual VL patient in Brazil, including diagnosis, treatment, and medical care, is estimated to be approximately $500 (US) (plus an additional $1470 (US) for secondary prophylaxis among VL patients with HIV) and lasts between seven and 20 days [49]. This is a high individual healthcare cost: yet, designing optimal surveillance that allows public health practitioners to understand and prevent VL is an incredibly valuable task socially and economically. These results and methods (applicable to any disease) can optimize disease data analysis and surveillance for the reduction of the systemic disease burden.

Using only VL incidence data and not introducing other data sources provides focus on what would be the outcome variable of a typical statistical analysis independently of any other predictors that may be introduced. Refitting models at multiple resolutions or scales assumes that the outcome, in this instance VL incidence, follows the same distribution in each scale and/or resolution. For example, using a lognormal regression model with two resolutions assumes that incidence at both resolutions follows a

lognormal distribution, which may not be correct. When analyzing municipality-resolution cases, not all states have distributions in the same family, and distributions following the same family have different parameterizations because of the likely differential importance of the underlying socio-environmental drivers. The latter point further motivates the use of Bayesian hierarchical models or other models, for instance statistical physics and/or information theoretic models, which are able to handle the information of scale and resolution controlling factors.

We show that the information theoretic RRIG can determine the amount of information needed to describe the data using different resolutions or scales. It can be used as an information theoretic tool for scaling (downscaling or upscaling, depending on the purpose) epidemiological data considering their value and underlying distributions.

An additional point of novelty is the use of the ZOIP and Gamma distributions to characterize VL incidence. Both distributions are uncommonly used for infectious disease incidence, despite closely fitting observed data. The ZOIP distribution offers the advantage of specifically fitting high frequencies of counts of one, describing single spurious cases. The Gamma distribution is advantageous for placing high probability on low values. More specifically into the statistical physics of disease ecology, the Gamma distribution has similarities to heavy tail distributions (for small shape and scale parameters) and ZOIP represents Poisson distributions highlighting local/random and medium-range disease dynamics. The higher statistical complexity (e.g. related to the number of parameters) of ZOIP reflects the random Poissonian nature of the disease with other factors, while the lower complexity of the Gamma reflects its more simple nature.

These analyses do not consider dependence on temporal resolution and scale although time and space for stochastic processes relate to each other. The data in this study include yearly case counts; having smaller time units such as months would allow for such consideration Additionally, distributions are assumed to

remain constant over the 11 years of observation considering the very minor variations in the inferred distributions that lead to consider VL dynamics at stationary state. Increases have been seen in VL cases over time [50], though case counts between 2000 and 2014 have remained more consistent compared to previous decades [51, 52], indicating that these results are not likely to be sensitive to this assumption. Populations over this time period by municipality generally showed small changes. The mean change in population by municipality was an increase of approximately 11% between 2004 and 2014, and the middle 90% of changes were between a 12% decrease and a 41% increase [33]. These considerations motivate extensions of this study to define the relationship between space and time for scale dependent processes.

Another assumption made in this study is the ability to fit a single probability distribution for VL incidence for the entire nation of Brazil. Since not all of the included states are considered endemic for VL [31], fitting a single distribution for incidence nationwide assumes that the same distribution can represent incidence in both endemic and non-endemic states. However, this consideration should be considered in the quantitative analyses that would follow from the results of this study. Other heterogeneities across the nation, such as affluence, urbanization, or climate, which may impact VL incidence, similarly are not considered for distribution fitting but should be accounted for during subsequent analyses.

The results of this study rely on the data collected. VL case data were collected through passive surveillance and notification to the Ministries of Health. It is commonly known that reported cases of infectious diseases only represent a portion of the total cases [53–55], commonly representing the most severe cases. This limits the accuracy of the data, and therefore distribution fitting, by the ability to report cases as well as the potential heterogeneous severity of VL cases. It is also likely that across locations in Brazil, amounts of underreporting of cases differ. The results of this study rely on the assumption that reported cases provide an adequate representation of disease burden. Furthermore, inclusion of both

endemic and non-endemic states in the analyses may lead to the inclusion of case data representing both typical VL incidence as well as atypical VL incidence. This could potentially affect distribution fitting if underlying processes leading to typical and atypical incidence differ.

A limitation of this study is the reliance on the criterion for determining differences when comparing distributions and algorithm used for determining best fitting distribution families and parameters. There are numerous methods for performing both tasks, and different methods may lead to slightly different conclusions. The methods of this study do, however, use assumption-free criteria in order to generate the results. A sensitivity analysis was conducted to determine if the number of samples drawn to generate the empirical state-resolution distribution described in section 2.2.1 using Equation 2 might impact RRIG values, and it was found that using 1,000; 2,000; 5,000; and 10,000 samples did not yield distinct difference in RRIG values and no differences in interpretations and conclusions.

Another important note is that this study used surveillance units of different sizes, examining aggregation of municipalities of differing land areas and populations and comparisons among states with different areas and populations. This results from using administrative districts, and still remains useful by using the units recorded in infectious disease surveillance. However, diseases know no political boundaries; yet, an ecosystem-based discretization to define homogeneous high resolution units would be preferable for surveillance such as one based on Digital Elevation Models from which to derive physical ecosystem boundaries that are relevant to disease spread. This would also help the control of diseases to assign to different political entities. A related topic of research is the existence of spatial autocorrelation in the data. Values of Moran’s I using municipality-resolution incidence nationwide showed strong evidence of spatial clustering. This implies that disease dynamics are non-local as already highlighted by differing fitted distributions across states, which is consistent with previous works [56, 57]. Subsequent analyses on VL in Brazil would benefit from the use of methods that account for spatial autocorrelation. For the purposes of distribution fitting, finding distributional families that most accurately characterize incidence

is of greater importance than determining a covariance structure that most accurately reflects autocorrelation. Determining clusters and covariance structures is an important component of analysis that follows the results of this study.

5. Conclusions

The choice of spatial resolution and scale in infectious disease research is shown to have an impact on results and conclusions of statistical and mathematical models. Finding sensitivity to the spatial resolution and spatial scale of VL surveillance data is of interest to both researchers and government officials for preparedness. Analyses using VL data should consider the findings of this study when planning analyses and controls related to disease processes or population incidence trajectories. Surveillance agencies should note that accurate surveillance by municipality is important because measuring incidence by state alone does not offer an equivalent characterization, and while there do exist small areas with incidences that can describes those of the others, nationwide surveillance at high resolution remains important to consider likely heterogeneity of processes contributing to VL burden. This applies to other diseases with incidences that depend on the scale and resolution of surveillance, which should be examined to assure whether this dependence does exist.

Acknowledgements: The authors thank the Brazilian Ministries of Health for allowing the use of the Visceral Leishmaniasis data for this study. The authors also acknowledge the resources of the Minnesota Supercomputing Institute for computational aid.

Author contributions:

Conceptualization: Joseph L Servadio, Gustavo Machado, Julio Alvarez, Matteo Convertino

Data Curation: Francisco Edilson de Ferreira Lima Júnior, Renato Vieira Alves

Formal Analysis: Joseph L Servadio

Funding Acquisition: Julio Alvarez, Matteo Convertino

Investigation: Francisco Edilson de Ferreira Lima Júnior, Renato Vieira Alves

Methodology: Joseph L Servadio

Project Administration: Matteo Convertino

Resources: Gustavo Machado, Julio Alvarez, Francisco Edilson de Ferreira Lima Júnior, Renato Vieira Alves

Software: Joseph L Servadio

Supervision: Matteo Convertino

Validation: Joseph L Servadio

Visualization: Joseph L Servadio

Writing – Original Draft Preparation: Joseph L Servadio, Gustavo Machado, Julio Alvarez, Matteo Convertino

Writing – Review & Editing: Joseph L Servadio, Gustavo Machado, Julio Alvarez, Francisco Edilson de Ferreira Lima Júnior, Renato Vieira Alves, Matteo Convertino

Competing interests: The authors do not have any competing interests.

References

1. Nsubuga P, White ME, Thacker SB, Anderson MA, Blount SB, Broome C V, et al. Public health surveillnace: A tool for targeting and monitoring interventions. In: Disease Control Priotiries in Developing Countries. 2nd ed. Washington, DC: The International Bank for Reconstruction and Development/The World Bank; 2006. p. 997–1015.
2. Hitchcock P, Chamberlain A, Van Wagoner M, Inglesby T V., O'Toole T. Challenges to Global Surveillance and Response to Infectious Disease Outbreaks of International Importance. Biosecurity Bioterrorism Biodefense Strateg Pract Sci [Internet]. 2007 Sep 28 [cited 2019 Jun 13];5(3):206–27. Available from: http://www.liebertpub.com/doi/10.1089/bsp.2007.0041
3. Pigott DM, Bhatt S, Golding N, Duda KA, Battle KE, Brady OJ, et al. Global distribution maps of the leishmaniases. Elife. 2014 Jun 27;3:e02851.
4. Linard C, Tatem AJ. Large-scale spatial population databases in infectious disease research. Int J Health Geogr [Internet]. 2012 [cited 2018 Jul 22];11:7. Available from: http://www.ij-healthgeographics.com/content/11/1/7
5. Levin SA. The Problem of Pattern and Scale in Ecology: The Robert H. MacArthur Award Lecture. Ecology [Internet]. 1992 Dec 1 [cited 2019 Apr 15];73(6):1943–67. Available from: http://doi.wiley.com/10.2307/1941447
6. Todd ECD. Challenges to global surveillance of disease patterns. Mar Pollut Bull [Internet]. 2006 Jan 1 [cited 2019 Jun 13];53(10–12):569–78. Available from: https://www.sciencedirect.com/science/article/pii/S0025326X06002931
7. Nsubuga P, Nwanyanwu O, Nkengasong JN, Mukanga D, Trostle M. Strengthening public health surveillance and response using the health systems strengthening agenda in developing countries. BMC Public Health [Internet]. 2010 Dec 3 [cited 2019 Jun 13];10(Suppl 1):S5. Available from: http://bmcpublichealth.biomedcentral.com/articles/10.1186/1471-2458-10-S1-S5
8. Piantadosi S, Byar DP, Green SB. The ecological fallacy. Am J Epidemiol. 1988;127(5):893–904.

9. Santos-Vega M, Martinez PP, Pascual M. Climate forcing and infectious disease transmission in urban landscapes: Integrating demographic and socioeconomic heterogeneity. Ann N Y Acad Sci. 2016;1382(1):44–55.

10. Mills HL, Riley S. The Spatial Resolution of Epidemic Peaks. PLoS Comput Biol. 2014 Apr 10;10(4):e1003561.

11. Pell B, Phan T, Rutter EM, Chowell G, Kuang Y. Simple multi-scale modeling of the transmission dynamics of the 1905 plague epidemic in Bombay. Math Biosci [Internet]. 2018 Jul [cited 2018 Jul 22];301:83–92. Available from: http://www.ncbi.nlm.nih.gov/pubmed/29673967

12. Jones SG, Kulldorff M. Influence of Spatial Resolution on Space-Time Disease Cluster Detection. PLoS One. 2012;7(10):e48036.

13. Mandelbrot B. How long is the coast of britain? Statistical self-similarity and fractional dimension. Science [Internet]. 1967 May 5 [cited 2019 Apr 16];156(3775):636–8. Available from: http://www.ncbi.nlm.nih.gov/pubmed/17837158

14. Peters EE. Fractal structures in the capital markets. Financ Anal J. 1989;45(4):32–7.

15. Camacho J, Guimerà R, Nunes Amaral LA. Robust Patterns in Food Web Structure. 2002 [cited 2019 Jan 5]; Available from: https://amaral.northwestern.edu/media/publication_pdfs/Camacho-2002-Phys.Rev.Lett.-88-228102.pdf

16. Kim H, Smith HB, Mathis C, Raymond J, Walker SI. Universal scaling across biochemical networks on Earth. Sci Adv. 2019 Jan 16;5(1):eaau0149.

17. Peng CK, Havlin S, Hausdorff JM, Mietus JE, Stanley HE, Goldberger AL. Fractal mechanisms and heart rate dynamics. Long-range correlations and their breakdown with disease. J Electrocardiol [Internet]. 1995 [cited 2019 Jan 5];28 Suppl:59–65. Available from: http://www.ncbi.nlm.nih.gov/pubmed/8656130

18. Schneeberger A, Mercer CH, Gregson SAJ, Ferguson NM, Nyamukapa CA, Anderson RM, et al. Scale-free networks and sexually transmitted diseases: a description of observed patterns of sexual contacts in Britain and Zimbabwe. Sex Transm Dis [Internet]. 2004 Jun [cited 2019 Jan

5];31(6):380–7. Available from: http://www.ncbi.nlm.nih.gov/pubmed/15167650

19. Small M, Walker DM, Tse CK. Scale-Free Distribution of Avian Influenza Outbreaks. Phys Rev Lett [Internet]. 2007 Oct 30 [cited 2019 Jan 5];99(18):188702. Available from: https://link.aps.org/doi/10.1103/PhysRevLett.99.188702

20. Convertino M, Liu Y, Hwang H. Optimal surveillance network design: a value of information model. Complex Adapt Syst Model [Internet]. 2014 Dec 27 [cited 2019 Apr 15];2(1):6. Available from: https://casmodeling.springeropen.com/articles/10.1186/s40294-014-0006-8

21. Ozgen VC, Kong W, Blanchard AE, Liu F, Lu T. Spatial interference scale as a determinant of microbial range expansion. Sci Adv. 2018 Nov 21;4(11):eaau0695.

22. Anversa L s., Tiburcio MGS, Richini-Pereira V nia B, Ramirez LE. Human leishmaniasis in Brazil: A general review. Vol. 64, Revista da Associacao Medica Brasileira. Associacao Medica Brasileira; 2018. p. 281–9.

23. World Health Organization. Leishmaniasis [Internet]. Fact Sheets. 2018 [cited 2018 Jul 22]. Available from: http://www.who.int/en/news-room/fact-sheets/detail/leishmaniasis

24. Bruhn FRP, Morais MHF, Cardoso DL, Bruhn NCP, Ferreira F, Rocha CMBM. Spatial and temporal relationships between human and canine visceral leishmaniases in Belo Horizonte, Minas Gerais, 2006–2013. Parasit Vectors [Internet]. 2018 Dec 28 [cited 2018 Jul 22];11(1):372. Available from: http://www.ncbi.nlm.nih.gov/pubmed/29954428

25. Killick-Kendrick R. The biology and control of Phlebotomine sand flies. Clin Dermatol [Internet]. 1999 May 1 [cited 2018 Jul 31];17(3):279–89. Available from: https://www.sciencedirect.com/science/article/pii/S0738081X99000462?via%3Dihub

26. Werneck GL. Visceral leishmaniasis in Brazil: rationale and concerns related to reservoir control. Rev Saude Publica [Internet]. 2014 Oct [cited 2018 Jul 22];48(5):851–6. Available from: http://www.ncbi.nlm.nih.gov/pubmed/25372177

27. Maia-Elkhoury ANS, Alves WA, Sousa-Gomes ML de, Sena JM de, Luna EA. Visceral leishmaniasis in Brazil: trends and challenges. Cad Saude Publica [Internet]. 2008 Dec [cited 2018

Jul 22];24(12):2941–7. Available from: http://www.ncbi.nlm.nih.gov/pubmed/19082286

28. Druzian AF, Souza AS de, Campos DN de, Croda J, Higa MG, Dorval MEC, et al. Risk Factors for Death from Visceral Leishmaniasis in an Urban Area of Brazil. Carvalho EM, editor. PLoS Negl Trop Dis [Internet]. 2015 Aug 14 [cited 2018 Jul 22];9(8):e0003982. Available from: http://www.ncbi.nlm.nih.gov/pubmed/26274916

29. World Health Organization. Leishmaniasis - Magnitude of the problem [Internet]. WHO. World Health Organization; 2014 [cited 2018 Jul 22]. Available from: http://www.who.int/leishmaniasis/burden/magnitude/burden_magnitude/en/

30. Ministério da Saúde Secretaria de Vigilância em Saúde Coordenação-Geral de Desenvolvimento da Epidemiologia emServiçosBrasil. Guia de Vigilância em Saúde : volume único [recurso eletrônico] . 3rd ed. 2019 [cited 2020 Apr 16]. 512–513 p. Available from: http://bvsms.saude.gov.br/bvs/publicacoes/guia_vigilancia_saude_3ed.pdf

31. Ministério da Saúde Secretaria de Vigilância em Saúde. Casos confirmados de Leishmaniose Visceral, Brasil, Grandes Regiões e Unidades Federadas. 1990 a 2018 [Internet]. 2019 [cited 2020 Apr 16]. Available from: https://www.saude.gov.br/images/pdf/2019/outubro/14/LV-Casos.pdf

32. Machado G, Alvarez J, Bakka HC, Perez A, Donato LE, de Ferreira Lima Júnior FE, et al. Revisiting area risk classification of visceral leishmaniasis in Brazil. BMC Infect Dis. 2019 Dec 3;19(1):2.

33. Instituto Brasileiro de Geografia Estastistica. Population Estimates [Internet]. 2018 [cited 2018 Aug 1]. Available from: https://www.ibge.gov.br/estatisticas-novoportal/sociais/populacao/9103-estimativas-de-populacao.html?edicao=17283&t=downloads

34. Alshkaki RSA. On the Zero-One Inflated Poisson Distribution. Int J Stat Distrib Appl. 2016 Dec 102(4):42–8.

35. Narula SC, Wellington JF. The Minimum Sum of Absolute Errors Regression: A State of the Art Survey. Int Stat Rev / Rev Int Stat~~ [Internet]~~. 1982 Dec~~ [cited 2020 Mar 3]~~;50(3):317.~~ Available from: https://www.jstor.org/stable/1402501~~

36. Kirkpatrick S, Gelatt CD, Vecchi MP. Optimization by simulated annealing. Science (80-). 1983;220(4598):671–80.

37. Cover TM, Thomas JA. Elements of Information Theory. 2nd ed. Hoboken, NJ: John Wiley & Sons; 2006.

38. Convertino M, Muñoz-Carpena R, Kiker GA, Perz SG. Design of optimal ecosystem monitoring networks: hotspot detection and biodiversity patterns. Stoch Environ Res Risk Assess [Internet]. 2015 May 14 [cited 2019 Apr 15];29(4):1085–101. Available from: http://link.springer.com/10.1007/s00477-014-0999-8

39. R Core Team. R: A language and environment for statistical computing [Internet]. Vienna, Austria: R foundation for statistical computing; 2019. Available from: https://www.r-project.org

40. Gillespie CS. Fitting heavy tailed distributions: The powerlaw package. J Stat Softw. 2015 Feb 1;64(2):1–16.

41. Sevá A da P, Mao L, Galvis-Ovallos F, Tucker Lima JM, Valle D. Risk analysis and prediction of visceral leishmaniasis dispersion in São Paulo State, Brazil. Carvalho EM, editor. PLoS Negl Trop Dis [Internet]. 2017 Feb 6 [cited 2019 Feb 11];11(2):e0005353. Available from: https://dx.plos.org/10.1371/journal.pntd.0005353

42. Campos R, Santos M, Tunon G, Cunha L, Magalhães L, Moraes J, et al. Epidemiological aspects and spatial distribution of human and canine visceral leishmaniasis in an endemic area in northeastern Brazil. Geospat Health [Internet]. 2017 May 11 [cited 2019 Feb 11];12(1):503. Available from: http://www.ncbi.nlm.nih.gov/pubmed/28555473

43. Afonso MM dos S, Chaves SA de M, Magalhães M de AFM, Gracie R, Azevedo C, de Carvalho BM, et al. Ecoepidemiology of American Visceral Leishmaniasis in Tocantins State, Brazil: Factors Associated with the Occurrence and Spreading of the Vector Lutzomyia (Lutzomyia) longipalpis (Lutz & Neiva, 1912) (Diptera: Psychodidae: Phlebotominae). In: The Epidemiology and Ecology of Leishmaniasis [Internet]. InTech; 2017 [cited 2019 Feb 11]. Available from: http://www.intechopen.com/books/the-epidemiology-and-ecology-of-

leishmaniasis/ecoepidemiology-of-american-visceral-leishmaniasis-in-tocantins-state-brazil-factors-associated-with

44. Reis LL dos, Balieiro AA da S, Fonseca FR, Gonçalves MJF, Reis LL dos, Balieiro AA da S, et al. Leishmaniose visceral e sua relação com fatores climáticos e ambientais no Estado do Tocantins, Brasil, 2007 a 2014. Cad Saude Publica [Internet]. 2019 Jan 10 [cited 2019 Feb 11];35(1). Available from: http://www.scielo.br/scielo.php?script=sci_arttext&pid=S0102-311X2019000105004&lng=pt&tlng=pt

45. Tejedor A, Singh A, Zaliapin I, Densmore AL, Foufoula-Georgiou E. Scale-dependent erosional patterns in steady-state and transient-state landscapes. Sci Adv. 2017 Sep 1;3(9):e1701683.

46. Lima ÁLM, de Lima ID, Coutinho JF V, de Sousa ÚPST, Rodrigues MAG, Wilson ME, et al. Changing epidemiology of visceral leishmaniasis in northeastern Brazil: a 25-year follow-up of an urban outbreak. Trans R Soc Trop Med Hyg~~ [Internet]~~. 2017~~ [cited 2019 Feb 11]~~;111(10):440–7. ~~Available from: http://www.ncbi.nlm.nih.gov/pubmed/29394411~~

47. Roy M, Zinck RD, Bouma MJ, Pascual M. Epidemic cholera spreads like wildfire. Sci Rep ~~[Internet]~~. 2014 Jan 15~~ [cited 2019 Nov 21]~~;4:3710. ~~Available from: http://www.ncbi.nlm.nih.gov/pubmed/24424273~~

48. Kinsley AC, Patterson G, VanderWaal KL, Craft ME, Perez AM. Parameter values for epidemiological models of foot-and-mouth disease in Swine. Front Vet Sci. 2016 Jun 1;3(JUN).

49. de Carvalho IPSF, Peixoto HM, Romero GAS, de Oliveira MRF. Cost of visceral leishmaniasis care in Brazil. Trop Med Int Heal~~ [Internet]~~. 2017 Dec 1~~ [cited 2019 Feb 11]~~;22(12):1579–89. ~~Available from: http://doi.wiley.com/10.1111/tmi.12994~~

50. Reis LL dos, Balieiro AA da S, Fonseca FR, Gonçalves MJF. Changes in the epidemiology of visceral leishmaniasis in Brazil from 2001 to 2014. Rev Soc Bras Med Trop~~ [Internet]~~. 2017 Sep ~~[cited 2019 Feb 11]~~;50(5):638–45. ~~Available from: http://www.ncbi.nlm.nih.gov/pubmed/29160510~~

51. World Health Organization. Number of cases of visceral leishmaniasis reported - Data by country

[Internet]. WHO. World Health Organization; 2018 [cited 2019 Feb 11]. Available from: http://apps.who.int/gho/data/node.main.NTDLEISHVNUM?lang=en

52. World Health Organization. Brazil [Internet]. [cited 2019 Feb 11]. Available from: https://www.who.int/leishmaniasis/resources/BRAZIL.pdf

53. Wheeler JG, Sethi D, Cowden JM, Wall PG, Rodrigues LC, Tompkins DS, et al. Study of infectious intestinal disease in England: Rates in the community, presenting to general practice, and reported to national surveillance. Br Med J. 1999 Apr 17;318(7190):1046–50.

54. Undurraga EA, Halasa YA, Shepard DS. Use of Expansion Factors to Estimate the Burden of Dengue in Southeast Asia: A Systematic Analysis. PLoS Negl Trop Dis. 2013;7(2).

55. Singh SP, Reddy DCS, Rai M, Sundar S. Serious underreporting of visceral leishmaniasis through passive case reporting in Bihar, India. Trop Med Int Heal. 2006 Jun;11(6):899–905.

56. Sadeq M. Spatial patterns and secular trends in human leishmaniasis incidence in Morocco between 2003 and 2013. Infect Dis Poverty. 2016;5(1).

57. Dewan A, Abdullah AYM, Shogib MRI, Karim R, Rahman MM. Exploring spatial and temporal patterns of visceral leishmaniasis in endemic areas of Bangladesh. Trop Med Health. 2017 Nov 15;45(1).

Supplemental material captions

S1 Table. Frequency of incidence values by municipality within each state and by state for the nation.

S2 Table. Best fitting parameterizations for each considered distribution and associated sum of absolute error (SAE) values.

S3 ~~Table~~File. Original data with annual Visceral Leishmaniasis ~~case counts and population~~incidence by municipality in Brazil, 2004 ~~-~~– 2014 with R code used for analysis.

UNIVERSITY OF MINNESOTA

Twin Cities Campus

Division of Environmental Health Sciences
School of Public Health

Suite 1260 Mayo
420 Delaware street SE
Minneapolis, MN 55455

16 April 2020

Dear Dr. Carvalho and reviewers,

Thank you for taking the time to review our manuscript and for giving us the opportunity to submit a revised version. We thank the reviewers for the helpful feedback on our study. Our manuscript has been modified and improved based on the comments. Several points were clarified in the manuscript based on the reviewers' comments, including some methodological decisions and clarifications as well as some important topics for the discussion section.

The reviewer comments are seen italicized, and responses to specific comments are found directly below relevant comments, indented.

Reviewer #1*: This is a very relevant study that addresses the effect of spatial scales on visceral leishmaniasis surveillance data from Brazil. The manuscript is very well written and understandable. The methods are adequately explained, although some minor details can be added to improve reproducibility.*

In infectious disease modelling, fitting disease data to a known statistical distribution is a common first step of the analyses, but oftentimes it is overlooked. The first great contribution of this work to VL modellers is summarised in table 2 – the fitted distributions for country and state-level surveillance datasets. I do not recall seeing the gamma distribution in other VL studies, which usually apply the Poisson distribution. This has the potential to become an important reference for VL modelling exercises in Brazil.

The second main contribution is the effect of spatial scale and the importance of choosing the right dataset for your research question. This has been explored before in theory, but to the best of my knowledge this is the first time I see these scale comparisons in VL data from Brazil.

I recommend the acceptance of the manuscript after some minor details are clarified, specifically:

L125: Explain why the Federal District was not included in the dataset.

> This study largely aimed to compare distributional fits at spatial resolutions, using incidence by municipality and by state. Because the Federal District is not

a state with multiple municipalities, Visceral Leishmaniasis incidence cannot be aggregated to allow municipality-resolution and state-resolution incidence to be calculated. This clarification has been added to the text on line 133 of the revised manuscript.

L131: Give further info on exclusion criteria for municipalities. Were they excluded due to missing population data only or disease data as well?

Excluded municipalities were not present in the IBGE population data. This sentence was rewritten to make this clearer, and appears on line 140 of the revised manuscript.

L222: It would be very relevant, for reproducibility, if the R code used in the analyses were uploaded as supporting information or uploaded to a code repository such as GitHub.

The third supplemental material, originally containing the original data, has been replaced with S3_File.zip. This contains a folder with the original incidence data as well as the R code used for these analyses.

L289: "different processes driving disease occurrence" – this made me think about the differences between disease occurrence and disease notification, which is the actual data that was analysed. Your discussion might also include comments on how underreporting of VL affect these analyses. It is very likely that underreporting varies among Brazilian states.

This is a very important note. This fact is very present in infectious disease literature. A paragraph has been added to the discussion highlighting that this study uses reported cases, which represent a subset of the true number of cases. This paragraph begins on line 418 of the revised document.

Fig. 1: In the map of Brazilian States there are some extra lines appearing where they should not, more specifically inside the states MS, GO, SP, ES and PE. This is probably an artefact of the shapefile that was used, it should be corrected.

We appreciate catching this detail. This has been fixed.

Reviewer #2*: I thank the authors for this work on an interesting subject. I suggest minor revision according to these comments:*
Introduction section:
1. "Urban area" Lines 98 and 101: Please check !

The citations associated with the statements indicate that Visceral Leishmaniasis has a canine reservoir in Brazil and that the disease has been seen in urban settings and is associated with urbanization in recent decades, in contrast to having been seen in rural settings previously. The wording has changed to

reflect that canine reservoirs are not exclusively in urban settings and that the recent shift to greater incidence in urban settings occurred since 1980 rather than the most recent years.

2. Authors don't precise Leishmania species caused VL in Brazil.

Cases of VL in Brazil have been attributed to *L. infantum*. This has been added on line 90 of the revised manuscript.

MM section:
1. Data collection: Please add subtitle for the first paragraph

This has been added.

2. 22 Brazilian states were included; please add data about epidemiological status: endemic/non endemic state!

Classifications of states as endemic/non endemic according to the Brazilian Ministries of Health, with citations, have been added, starting on line 104 of the revised manuscript as well as in Table 2.

Discussion section:
Authors must discuss according to other parameters: epidemiological status of the study area, type of infection....

This point of epidemiological status has been included. Similarly, other heterogeneities across Brazil are not accounted for in the distribution fitting process, but should be considered in any modeling studies that follow. This has been added in the paragraph starting on line 409 of the revised manuscript as well as on lines 424-428.

The point of the type of infection connects with the other reviewer's comment regarding underreporting of cases, as the most severe cases are less commonly underreported. The severity of infection has been added as a point describing underreporting on lines 446-448 of the revised manuscript.

In addition to these specific comments, the manuscript has been checked for any spelling/grammatical errors as well as citations and other formatting matters. We appreciate your consideration of our revised manuscript and hope to hear from you soon.

Thank you,

Joseph L Servadio
