# Supplementary material for: Information differences across spatial resolutions and scales for disease surveillance and analysis: The case of Visceral Leishmaniasis in Brazil
Source: PLoS One. 2020 Jul 17;15(7):e0235920. doi: 10.1371/journal.pone.0235920 (PMC7367469; doi:10.1371/journal.pone.0235920)
Supplement: Attachment — Submitted filename: Servadio_ResponseToReviewers_Jun2020.pdf [file pone.0235920.s006.pdf]

UNIVERSITY OF MINNESOTA

Twin Cities Campus

Division of Environmental Health Sciences
School of Public Health

Suite 1260 Mayo
420 Delaware street SE
Minneapolis, MN 55455

5 Jun 2020

Dear Dr. Carvalho and reviewers,

Thank you for taking the time to review our manuscript and for giving us the opportunity to submit a revised version. We thank the reviewers for the additional helpful feedback on our study. Our manuscript has been modified and improved based on the comments. Several points were clarified in the manuscript based on the reviewers' comments, including some methodological decisions and clarifications as well as some important topics for the discussion section.

The reviewer comments are seen italicized, and responses to specific comments are found directly below relevant comments, indented.

Reviewer #2: The authors have adequately addressed my comments. This version of the manuscript is now acceptable for publication.

> We appreciate the reviewer's attention to our revision.

Reviewer #3: This study is a good study and it is recommended to be accepted for publish. some comments:
It is better to work on the resolution of the maps.

> All figures have an improved resolution.

Specify the north and south directions on the map.

> The maps in Figures 2 and 4 contain a compass arrow pointing to North in the lower left corner of each map. Figure 1 does not contain this because it is a conceptual diagram; specific details of the orientation of Brazil are not crucial to its interpretation.

In the introduction, the mortality rate of visceral leishmaniasis in the world and Brazil should be mentioned.

> This has been added. The global mortality rate, represented via estimates of total estimated cases and deaths, is reported on lines 102 and 103 of the revised manuscript. The estimated mortality rate in Brazil is reported on lines 107-110.

Reviewer #4: I would like to thank the authors for this interesting work on VL. Please find my detailed comments below:

The Modifiable Areal Unit Problem (MAUP) is a common problem for analyzing spatial data, which I think the authors have partially engaged by mentioning about the scale invariance problem. However, it is still unclear from the introduction as to where the research gap lies, when it is a common knowledge that changing the scale is highly likely to change the results or findings of a spatial analysis. The objective of the work was outlined well but the motivation of the work requires more clarity.

> This study's relevance to the MAUP is related to that to ecological fallacy. Few studies have quantified this, as this concept is intuitive. This study offers an example of a real quantification of this issue. These points are included in the introduction on lines 63-66 and in the discussion on lines 366-368 of the revised manuscript.
>
> The second motivation and objective of this study was to examine scale invariance, which differs in that the size of the entire area considered is examined, while keeping the areal units constant. This is motivated by the ability to use a subset of the nation of Brazil to describe the nation's VL burden, as described in the introduction.

Line 145 to 146: Any reason why this decision was taken?

> Previous studies exploring this have done so by first posing a substantive research question and then following up by repeating analyses. The argument made in this manuscript is that, while providing some insight, the repeated analysis is subject to the assumptions of the model. Because this study aimed to address the question of scale/resolution sensitivity as the primary objective, a model using other covariates was not wanted. Fitting the distributional fit represents the simplest way of describing disease incidence and would directly relate to either a stochastic node of a mathematical model or a link function of a statistical model. An additional sentence emphasized this point on lines 163-166 of the revised manuscript.

Line 152: What exploratory analyses were conducted and what criteria for suitability were set for choosing the distributions in Table 1? I see ZIP and ZOIP are used, which is understandable given the count nature of the data. But it is unclear as to why the exponential, gamma etc. models were necessary. This part of the manuscript could be a little tricky to deal with, as the justifications of the selection of the distributions need to be provided without making the methods too technical for the readers to understand. Again, the justification needs to be clear enough so that any readers with a geostatistical background can understand the rationale behind the selection and can benefit from this work.

The exploratory analyses were primarily visualizations and examining summary statistics such as quantiles and moments. Suitability refers to a distribution having a nonnegative support. The purpose of selecting a variety of distributions was to consider many common distributional families to explore the possibility of one providing the closest fit to the data. These points were made more explicit in the text and appear on lines 172-173 of the revised manuscript.

Line 165: How was "()" or "the probability of observing an incidence o f cases per 100,000 person-years" determined? Did the process involve the computation of any expected number of cases? If yes, what method was applied for the computation?

The values of p(x) were calculated using the formulas in Table 1. These were evaluated through base R functions such as rpois(), runif(), etc, based on the distributional family being evaluated. In the cases of the Power Law distribution, the 'poweRlaw' package was used as cited at the end of the methods section.

Line 187: The authors have repeatedly mentioned this (or similar) term "inferential conclusions". I think it is better to clearly outline what this term entails for the readers to understand the methods better.

We appreciate this point. The intended message is that the finding that the scale and resolution choices can change the distributional fit of incidence implies that any statistical or mathematical models using this data could suffer from similar sensitivity given choices of scale and resolution. The wording has been changed throughout the manuscript to explicitly state this every time with consistent wording. Notable changes include those on lines 210-213 and 437-438 of the revised manuscript.

The left hand side of Eq2 is absent

A value has been added for the left hand side. It is also referenced in a sentence added to the end of the paragraph (line 232) to clarify the methods used.

Line 223: Any justification for this 5% threshold? Please include in the text for strengthening your selection.

The 5% threshold was decided a priori, and not based on previous use. This has been added on line 251 of the revised manuscript and a sentence has been added to the discussion on lines 452-464 that states this explicitly.

Results have been presented well but the discussion needs to engage with the issue of spatial heterogeneity that has not been incorporated into the analysis. For example, Fig 1 shows the distribution of VL cases can possibly exhibit spatial clustering and even

spatial autocorrelation. Under these circumstances, there is a good possibility that the incidence rates might not match across different scales, as the disease burden is not distributed equally across all the municipalities.

> The discussion of spatial heterogeneity has been expanded to incorporate these points more directly. This was noted in the last paragraph of the discussion section. It has been made into its own paragraph with an addition to make this point more explicit.

In addition to these specific comments, the manuscript has been checked for any spelling/grammatical errors as well as citations and other formatting matters. We appreciate your consideration of our revised manuscript and hope to hear from you soon.

Thank you,

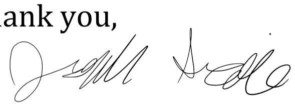

Joseph L Servadio
